# Identification of Chemotypic Markers in Three Chemotype Categories of Cannabis Using Secondary Metabolites Profiled in Inflorescences, Leaves, Stem Bark, and Roots

**DOI:** 10.3389/fpls.2021.699530

**Published:** 2021-07-01

**Authors:** Dan Jin, Philippe Henry, Jacqueline Shan, Jie Chen

**Affiliations:** ^1^Department of Biomedical Engineering, University of Alberta, Edmonton, AB, Canada; ^2^PBG BioPharma Inc., Leduc, AB, Canada; ^3^Egret Bioscience Ltd., West Kelowna, BC, Canada; ^4^Lighthouse Genomics Inc., Salt Spring Island, BC, Canada; ^5^Department of Electrical and Computer Engineering, University of Alberta, Edmonton, AB, Canada

**Keywords:** cannabis, THC, CBD, chemotypes, markers, secondary metabolites, plant parts

## Abstract

Previous chemotaxonomic studies of cannabis only focused on tetrahydrocannabinol (THC) dominant strains while excluded the cannabidiol (CBD) dominant strains and intermediate strains (THC ≈ CBD). This study investigated the utility of the full spectrum of secondary metabolites in different plant parts in three cannabis chemotypes (THC dominant, intermediate, and CBD dominant) for chemotaxonomic discrimination. Hierarchical clustering, principal component analysis (PCA), and canonical correlation analysis assigned 21 cannabis varieties into three chemotypes using the content and ratio of cannabinoids, terpenoids, flavonoids, sterols, and triterpenoids across inflorescences, leaves, stem bark, and roots. The same clustering results were obtained using secondary metabolites, omitting THC and CBD. Significant chemical differences were identified in these three chemotypes. Cannabinoids, terpenoids, flavonoids had differentiation power while sterols and triterpenoids had none. CBD dominant strains had higher amounts of total CBD, cannabidivarin (CBDV), cannabichromene (CBC), α-pinene, β-myrcene, (−)-guaiol, β-eudesmol, α-eudesmol, α-bisabolol, orientin, vitexin, and isovitexin, while THC dominant strains had higher total THC, total tetrahydrocannabivarin (THCV), total cannabigerol (CBG), camphene, limonene, ocimene, sabinene hydrate, terpinolene, linalool, fenchol, α-terpineol, β-caryophyllene, trans-β-farnesene, α-humulene, trans-nerolidol, quercetin, and kaempferol. Compound levels in intermediate strains were generally equal to or in between those in CBD dominant and THC dominant strains. Overall, with higher amounts of β-myrcene, (−)-guaiol, β-eudesmol, α-eudesmol, and α-bisabolol, intermediate strains more resemble CBD dominant strains than THC dominant strains. The results of this study provide a comprehensive profile of bioactive compounds in three chemotypes for medical purposes. The simultaneous presence of a predominant number of identified chemotype markers (with or without THC and CBD) could be used as chemical fingerprints for quality standardization or strain identification for research, clinical studies, and cannabis product manufacturing.

## Introduction

Cannabis is a complex herbal medicine containing several classes of secondary metabolites, including cannabinoids, terpenoids, flavonoids, and steroids among 545 identified compounds (Turner et al., 1980; ElSohly and Slade, 2005; Ross et al., 2005; ElSohly and Gul, 2014; Russo and Marcu, 2017; Pollastro et al., 2018; Jin et al., 2020). For medical applications, researchers widely adopt a chemotaxonomic perspective that describes three chemotypes (chemical phenotypes) based on the content of two major cannabinoids: psychoactive tetrahydrocannabinol (THC) and non-psychoactive cannabidiol (CBD) (Small and Beckstead, 1973; Turner et al., 1979; Mandolino et al., 2003; de Meijer et al., 2009). THC dominant strains have a ratio of THC/CBD > 1, intermediate strains have THC/CBD ≈ 1, and CBD dominant strains have THC/CBD < 1. Although most clinical studies focus on THC and CBD, increasing amounts of evidence show that whole plant extract has additional benefits when compared to single cannabinoids. In one study, whole cannabis extract was more effective in inducing cancer cell death than applying pure THC on cancer cell lines (Baram et al., 2019). In addition, individual cannabis extracts with similar amounts of THC produced significantly different effects on the survival of specific cancer cells, and specific cannabis extracts may selectively and differentially affect different cancer cells lines (Baram et al., 2019). In another study, extracts from five strains with similar CBD concentrations had different anticonvulsant properties in mice (Berman et al., 2018). These studies suggest that there may exist therapeutic-enhancing interactions or synergistic effects amongst cannabinoids as well as between cannabinoids and other secondary metabolites, known as the “entourage effect” (McPartland and Russo, 2001; Russo, 2011; Blasco-Benito et al., 2018). It is therefore essential to have a comprehensive, full spectrum metabolic fingerprinting of secondary metabolites in cannabis materials for research and clinical studies. Previous research also focused on female inflorescences, however, each part of the plant has a wide range of indications, primarily related with pain and inflammation, as ancient herbal medicines in various cultures (Smith and Stuart, 1911; Brand and Wiseman, 2008; Brand and Zhao, 2017; Ryz et al., 2017). Our previous study profiled cannabinoids, terpenoids, flavonoids, sterols, and triterpenoids, not only in cannabis inflorescences, but also in leaves, stem bark, and roots (Jin et al., 2020). By profiling these compounds in each cannabis plant part and associating them with therapeutic benefits, cannabis plant material that is currently treated as waste has potential to be developed into natural health products or medications.

Cannabis classification is a fundamental requirement for future medical research and applications, and it is best enabled through an overview of the class and content of potentially therapeutic secondary metabolites in each plant part. Currently, researchers attempted to discriminate and identify the chemical differences between the categories of “Sativa” (narrow-leaflet drug, NLD) and “Indica” (wide-leaflet drug, WLD) (Fischedick et al., 2010; Hazekamp and Fischedick, 2012; Hazekamp et al., 2016). Results of the chemotaxonomic separation of “Sativa” and “Indica” were mixed, and THC and CBD concentrations appeared to have no differentiation value. However, certain terpenoids were more prominent in some strains than others (Hillig, 2005b; Fischedick et al., 2010; Hazekamp and Fischedick, 2012; Fischedick, 2015, 2017; Hazekamp et al., 2016; Jin et al., 2017; McPartland and Guy, 2017). The mixed results in the current body of literature may be due to experimental design shortcomings. Firstly, the vernacular terminology (“Sativa” and “Indica”) is inadequate for medical applications due to the misuse of the botanical nomenclature, extensive cross-breeding, and unreliable labeling during unrecorded hybridization (McPartland, 2017). Secondly, samples in most classification studies were collected from disparate sources (Fischedick et al., 2010; Hazekamp et al., 2016) and are subject to inconsistent environmental factors during the growth phases (Aizpurua-Olaizola, 2016) and post-harvest treatment (Jin et al., 2019). Additionally, inappropriate sample preparation and extraction procedures during laboratory analysis may affect classification results (Jin et al., 2020). All these factors contribute to the variation in chemical profiles of the final products, which in turn leads to inconsistent results and poor classification accuracy. More accurate classification results are obtainable when plants are grown in a single location, under identical environmental conditions, and uniformly processed (McPartland, 2017).

The chemical profile of CBD dominant and intermediate strains, which have gained increasing attention due to CBD’s use as a therapeutic (Avraham et al., 2011; French et al., 2017; McGuire et al., 2018; Bloomfield et al., 2020), have not been studied or compared to THC dominant strains in the current literature. In this study, we used unsupervised hierarchical clustering and principal component analysis (PCA) as well as supervised canonical correlation analysis to test the goodness of fit between chemotype labeling (THC dominant, intermediate, and CBD dominant) and chemotypic variation of the full spectrum of secondary metabolites in various plant parts of 21 strains. This study also identifies chemotypic markers within each chemotype, which will facilitate strain selection for further clinical and research studies.

The objectives of this study are to:

1. investigate whether modern cannabis strains can be differentiated using a full spectrum of secondary metabolites in three chemotypes, including 14 cannabinoids, 45 terpenoids, 7 flavonoids, 3 sterols, and 3 triterpenoids, in inflorescences, leaves, stem bark, and roots;
2. investigate whether the secondary metabolites described above can differentiate strains into three chemotypes without leveraging THC and CBD data; and
3. identify chemotypic markers that can be leveraged to select and distinguish chemotypes.

## Materials and Methods

### Plant Material

**FIGURE 1:**
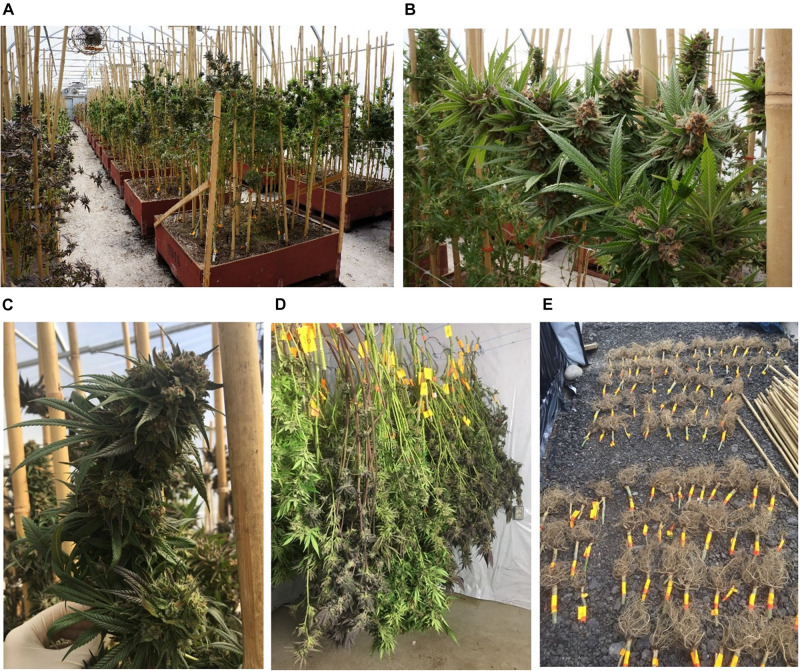
Cannabis grown in a commercial greenhouse. **(A–C)** Cannabis plants before harvest. **(D)** Whole cannabis plants were cut above the ground and hang to dry in a drying room. **(E)** Cannabis roots were individually labeled and dried in the drying room with the other plant parts.

In this project, 21 commercially available cannabis strains were grown in a commercial greenhouse (Figure 1) under a cannabis research license issued by Health Canada. Where possible, the reported ancestry (“Sativa-dominant,” “Indica-dominant,” or “hybrid”) was obtained from the Leafly online database^1^ or from the licensed cultivator providing the strain (Supplementary Table 1). Three to five cuttings per strain were rooted for 2 weeks, followed by vegetative growth under 24 h photoperiod for 2 months, and then flowered under 12 h photoperiod. After 2 months of flowering, the plants were harvested and hung to dry in a closed environment. Cannabis roots were removed and dried in the same room together with the other plant parts. Horticultural fans were used to maintain air circulation, and the temperature was kept under 35°C. The plants were dried for 7 days until the leaves and stems became brittle. At this time, the plants’ moisture content is usually below 10–15% (mg/mg%) (Potter, 2009; Caplan, 2018).

### Sample Preparation, Extraction, and Assay

A total of 82 plants representing 21 strains were harvested. Inflorescences, leaves (fan leaves), stem bark, and roots were separately collected for each plant and analyzed for the full spectrum of secondary metabolites. Sugar leaves (small leaves extending from the inflorescences) were treated as a part of the inflorescences. Samples were prepared and analyzed according to previously developed and validated methodologies (Jin et al., 2020). Five to eight flower heads (2–4 g) of each plant were pulverized with a SPEX Geno/Grinder homogenizer (SPEX SamplePrep, Canada). Dried leaf material was crushed using a mortar and pestle and sifted through a 1.18 mm sieve. Dried stem bark and root samples were ground with the SPEX Geno/Grinder homogenizer. For cannabinoids and terpenoids extraction, 400 mg of plant material was extracted with 20 mL methanol (with 100 μg/mL tridecane as an internal standard for mono- and sesquiterpenoids) by sonication for 20 min at room temperature. For cannabinoids, the extract was spiked with Δ^9^-THC-d_3_ (0.5 μg/mL) as an internal standard prior to LC-MS analysis. One aliquot of the extract was used to quantify mono- and sesquiterpenoids using GC-MS. For flavonoids extraction, 250 mg of the sample was extracted with 5 mL of ethanol, water, and hydrochloric acid at a 25:10:4 volume ratio. The extract was hydrolyzed in a 100°C water bath for 135 min. The tube was then repeatedly rinsed with methanol, and the rinses were combined with the extract in a 50 mL volumetric flask, which was filled to volume with methanol. For the flavonoids assay, HPLC was used with an UV detector at 350 nm for the quantification of seven flavonoids and MS detector for compound identification. For triterpenoids and sterols extraction, 1 g of dried sample was extracted with 20 mL ethyl acetate by sonication for 1 h, followed by maceration for one day at room temperature. The extract was spiked with cholesterol (50 μg/mL) as an internal standard prior to GC-MS analysis.

### Statistical Analysis

In total, 82 plants representing 21 strains were included in the following analysis. Cannabinoids were calculated as the sum of their neutral forms, metabolites (if applicable), and cannabinoid acids (multiplied by a factor converting acids into their corresponding neutral forms). For example, total THC = Δ^9^ - THC + Δ^8^ - THC + CBN (cannabinol, degradation product of THC) + 0.877 × tetrahydrocannabinolic acid (THCA), total CBD = CBD + 0.877 × cannabidiolic acid (CBDA), total cannabigerol (CBG) = CBG + 0.878 × cannabigerolic acid (CBGA), total cannabichromene (CBC) = CBC + 0.877 × cannabichromenic acid (CBCA), total tetrahydrocannabivarin (THCV) = THCV + 0.867 × tetrahydro-nabivarinic acid (THCVA), and total cannabidivarin (CBDV) = CBDV + 0.867 × cannabidivarinic acid (CBDVA); (Upton et al., 2014; Jin et al., 2020). Total cannabinoids was calculated as the sum of 14 cannabinoids. Total monoterpenoids (terpenoids with two isoprene units in the chemical structure) was the sum of the 29 monoterpenoids in Supplementary Table 2.5, and total sesquiterpenoids (terpenoids with three isoprene units) were calculated as the sum of the 16 sesquiterpenoids. Total terpenoids was the sum of total mono- and sesquiterpenoids. Total flavonoids was the sum of seven flavonoids after acid hydrolysis, including orientin, vitexin, isovitexin, quercetin, luteolin, kaempferol, and apigenin. Total sterols was the sum of campesterol, stigmasterol, and β-sitosterol. Total triterpenoids was the sum of β-amyrin, epifriedelanol, and friedelin. Compound ratios were calculated by dividing the content of one compound by the total content of that metabolite group. For example, the ratio of β-pinene was calculated as its absolute value divided by total terpenoids.

Secondary metabolites were quantified in each plant part. The following analyses were carried out only on the metabolites in the plant part where they were of highest levels among all plant parts. This distinction is made for isolating metabolites where they are present in sufficiently high concentrations (above 0.05%) to be of pharmacological interest (Russo, 2011). First, correlations were calculated between individual cannabinoids, terpenes, flavonoids, sterols, and triterpenoids. Because absolute values vary with environmental factors and relative proportions are more stable (Hillig, 2005a), compound ratios were used. Then, unsupervised (no preassigned categories as constraints) hierarchical clustering using Ward’s minimum variance method (Ward, 1963) and PCA (Jolliffe, 2002) were used to check within-strain and between-cluster variation. Finally, the data were subjected to supervised (with preassigned categories as constraints) canonical correlation analysis with preassigned chemotypes in Table 1. The full spectrum of secondary metabolites, without THC and CBD, were subjected to hierarchical clustering, PCA, and canonical correlation analysis to investigate whether the absence of THC and CBD data would affect differentiating strains into chemotypes.

**TABLE 1:** Preassigned chemotypes as the working groups for canonical correlation analysis.

Clusters	Number of strains	Strain codes as chemotypes
C1 (CBD dominant)	6	3-CBD, 4-CBD, 5-CBD, 6-CBD, 8-CBD, 10-CBD
C2 (Intermediate)	3	1-Intermediate, 2-Intermediate, 9-Intermediate
C3 (THC dominant)	12	11-THC, 12-THC, 13-THC, 14-THC, 15-THC, 16-THC, 18-THC, 19-THC, 20-THC, 21-THC, 22-THC, 23-THC

Canonical correlation analysis is also called canonical variates analysis, and is a multiple discriminant analysis that calculates the correlation between preassigned clusters and the set of covariates (chemical compounds in this study) describing the observations (Hotelling, 1936). The first canonical variable is the linear combination of the covariates that maximizes the multiple correlation between the clusters and the covariates. The second canonical variable is a linear combination uncorrelated with the first canonical variable that maximizes the multiple correlation. The analysis outputs a biplot with the first two canonical variables that provide maximum separation among the clusters. To identify marker metabolites that contribute most to the groupings, one-way ANOVA followed by Tukey honestly significant difference (HSD) *post hoc* test at the 0.05 significance level were used to determine whether significant differences exist between all clusters and each pair of clusters. Statistical analysis was performed with JMP 14.0.0.

## Results

### Secondary Metabolites Profiled in Cannabis Inflorescences, Leaves, Stem Bark, and Roots

Secondary metabolites profiled in inflorescences, leaves, stem bark, and roots are provided in Supplementary Table 9. Average total cannabinoids content from 82 plants of 21 strains decreased in order of inflorescences, leaves, stem bark, and roots, as shown in Supplementary Figure 1. Total cannabinoids were between 7.06 and 24.42% with an average of 15.90 ± 4.02% (SD) in inflorescences, between 0.95 and 4.28% with an average of 2.17 ± 0.71% in leaves, between 0.06 and 2.33% with an average of 0.58 ± 0.28% in stem bark, and less than 0.03% in roots (Supplementary Table 2.1). Total average cannabinoids content in inflorescences were 17.16 ± 4.60%, 14.98 ± 2.63%, and 13.96 ± 2.15% in THC dominant, intermediate, and CBD dominant strains, respectively (Supplementary Table 2.2). These values are typical for modern cannabis strains in North America and mostly agreed with reported values in the literature, which are generally between 5 and 25% (ElSohly and Gul, 2014; Fischedick, 2015; Hazekamp et al., 2016; Lynch et al., 2016; Jikomes and Zoorob, 2018; Richins et al., 2018). THC dominant strains had significantly higher concentrations of cannabinoids than the other two chemotypes (*p* = 0.0035). Total cannabinoids content in leaves and stem bark averaged from three chemotypes are summarized in Supplementary Tables 2.3, 2.4.

Average total terpenoids as the sum of mono- and sesquiterpenoids in the same population decreased in order of inflorescences, leaves, stem bark, and roots (Supplementary Figure 1). Total terpenoids in inflorescences was between 0.753 and 3.305% with an average of 1.509 ± 0.467%, in leaves between 0.035 and 0.197% with an average of 0.103 ± 0.032%, and in stem bark and roots less than 0.03% (Supplementary Table 2.1). Average total terpenoids content in inflorescences and leaves for the three chemotypes are summarized in Supplementary Tables 2.5, 2.6.

Average total flavonoids as the sum of orientin, vitexin, isovitexin, quercetin, luteolin, kaempferol, and apigenin was highest in leaves, lower in inflorescences, and less than 0.03% in stem bark and roots (Supplementary Figure 1). Total flavonoids in inflorescences were between 0.028 and 0.284% with an average of 0.091 ± 0.050%, and in leaves between 0.051 and 0.470% with an average of 0.188 ± 0.098% (Supplementary Table 2.1). Flavonoids exist in cannabis plants as both aglycones and conjugated glycosides and were estimated to be less than 1% in leaves (McPartland and Russo, 2001) The results of this study was congruent with this estimate, since the flavonoids were not converted to conjugated glycosides. All seven flavonoids were quantifiable in inflorescences in three chemotypes (Supplementary Table 2.7), while quercetin and kaempferol were below the quantification limit in leaves (Supplementary Table 2.8). All flavonoids identified in inflorescences and leaves were less than those reported in other studies (Flores-Sanchez and Verpoorte, 2008), possibly due to differences in strains and plant growth stage, since flavonoids content fluctuate with plant age (Vanhoenacker et al., 2002).

Total sterols content as the sum of three phytosterols, campesterol, stigmasterol, and β-sitosterol was highest in roots, lower in stem bark, and was less than 0.03% in inflorescences and leaves (Supplementary Figure 1). Total sterols content in roots was between 0.037 and 0.085% with an average of 0.066 ± 0.009%, and in stem bark was between 0.037 and 0.082% with an average of 0.055 ± 0.013% (Supplementary Table 2.1). Average total sterols content in stem bark and roots of the three chemotypes are summarized in Supplementary Tables 2.9, 2.10.

Total triterpenoids as the sum of β-amyrin, epifriedanol, and friedelin was highest in roots, lower in stem bark, and was less than 0.03% in inflorescences and leaves (Supplementary Figure 1). Total triterpenoids in stem bark was between 0.008 and 0.136% with an average of 0.039 ± 0.023%, in roots was between 0.080 and 0.275% with an average of 0.182 ± 0.043% (Supplementary Table 2.1). Average total triterpenoids content in stem bark and roots in the three chemotypes are summarized in Supplementary Tables 2.11, 2.12.

The distribution of secondary metabolites in each plant part agreed with conclusions from our last study (Jin et al., 2020). Correlation and classification analyses were performed only for metabolites in the plant part where they were present in the highest concentrations representative for that strain. For example, the average terpenoid content in leaves were low (0.103 ± 0.032%) compared to the levels in inflorescences (1.509 ± 0.467%), and only 15 mono- and sesquiterpenoids that were detected in inflorescences were above the quantification limit in leaves (Supplementary Table 2.6). In addition, the correlations between cannabinoids and terpenoids in leaves were like those in inflorescences, especially for the terpenoids that are abundant in both these two plant parts, including α-pinene, β-pinene, limonene, linalool, β-caryophyllene, trans-β-farnesene, α-humulene, trans-nerolidol, (−) guaiol, β-eudesmol, α-eudesmol, and α-bisabolol (Supplementary Figure 2 and Supplementary Table 8). As such, using the terpene profile in inflorescences was adequate for clustering purposes. Flavonoids in inflorescences and leaves were included in the analysis because quercetin and kaempferol were quantifiable in inflorescences but not in leaves. For sterols, the content and ratios of three sterols are similar between stem bark and roots. Because total sterols in roots (0.064–0.068%) are slightly higher than them in stem barks (0.052–0.059%), the sterol profiles in roots were used in the data analysis. Triterpenoid profile in roots were used because the content of total triterpenoids was above the threshold for pharmacological interest in all plant parts except in roots. To summarize, the most abundant secondary metabolites in individual plant parts were used in the statistical analysis for identifying differences between the three chemotypes. These metabolites were cannabinoids, terpenes, and flavonoids in inflorescences; flavonoids in leaves; and sterols and triterpenoids in roots (Supplementary Table 7).

### Correlation Analysis Between Secondary Metabolites

**FIGURE 2:**
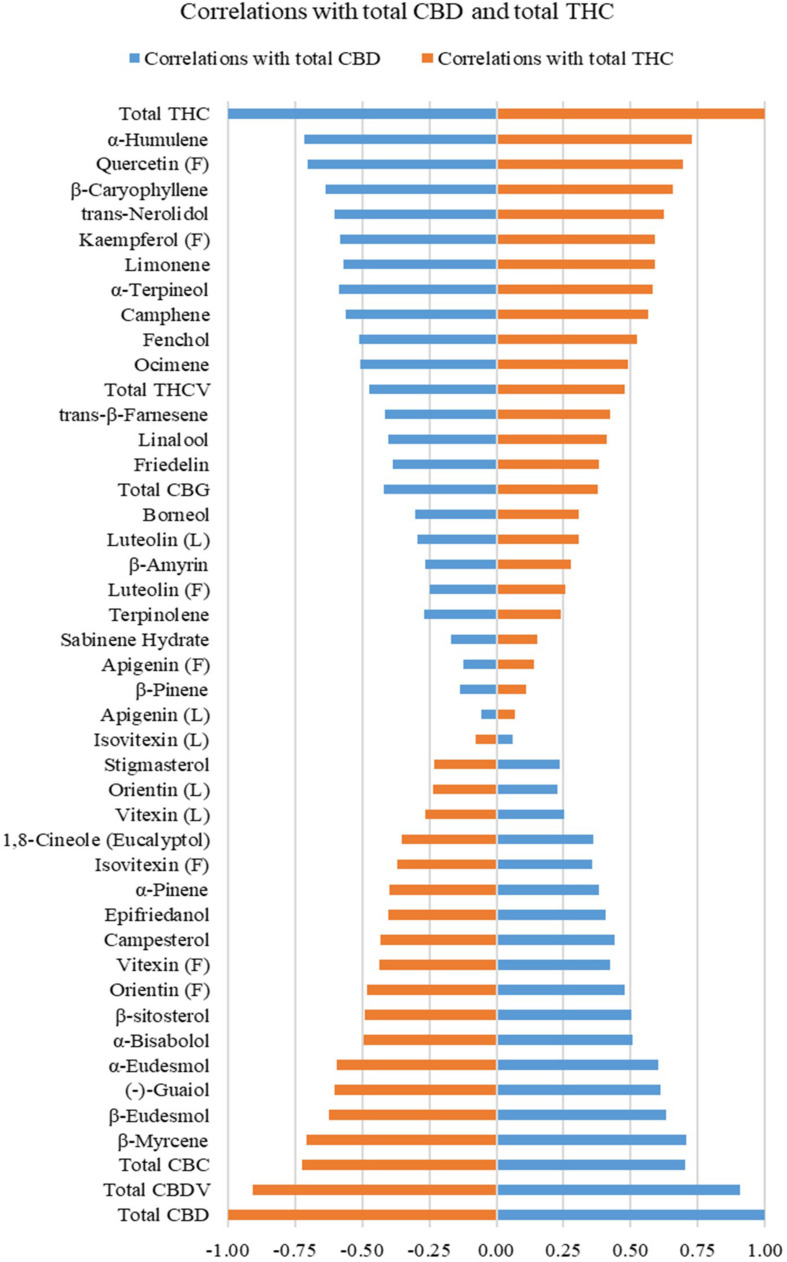
Correlations of total THC and total CBD with cannabinoids (in inflorescences), mono- and sesquiterpenoids (in inflorescences), flavonoids (in inflorescences and leaves), sterols (in roots), and triterpenoids (in roots) on quantifiable compounds using ratios. Flavonoids quantified in inflorescences are labeled (F), and flavonoids in leaf are labeled (L).

Correlations between total THC or total CBD with individual cannabinoids, terpenoids, flavonoids, sterols, and triterpenoids are plotted in Figure 2 and summarized in Supplementary Table 3. Calculations were performed on quantifiable compounds using ratios. Total THC was positively correlated with two cannabinoids (total CBG and total THCV), 10 monoterpenoids (α-terpineol, limonene, camphene, fenchol, linalool, ocimene, borneol, terpinolene, β-pinene, and sabinene hydrate), four sesquiterpenoids (α-humulene, β-caryophyllene, trans-nerolidol, and trans-β-farnesene), four flavonoids (quercetin and kaempferol in flowers, luteolin and apigenin in both inflorescences and leaves), and two triterpenoids (β-amyrin and friedelin). Total CBD was positively correlated with two cannabinoids (total CBDV and total CBC), three monoterpenoids (β-myrcene, 1,8-cineole (eucalyptol), α-pinene), four sesquiterpenoids (β-eudesmol, (−)-guaiol, α-eudesmol, α-bisabolol), three flavonoids (orientin, vitexin, isovitexin in both inflorescences and leaves), three sterols (campesterol, stigmasterol, β-sitosterol), and one triterpenoid (epifriedanol). Compounds that were positively correlated with THC were all negatively correlated with total CBD, and vice versa. The quantitative correlations are plotted in Supplementary Figure 3. Most compounds have similar correlations with total THC and total CBD when calculated using ratios and absolute values.

### Unsupervised Hierarchical Clustering

**FIGURE 3:**
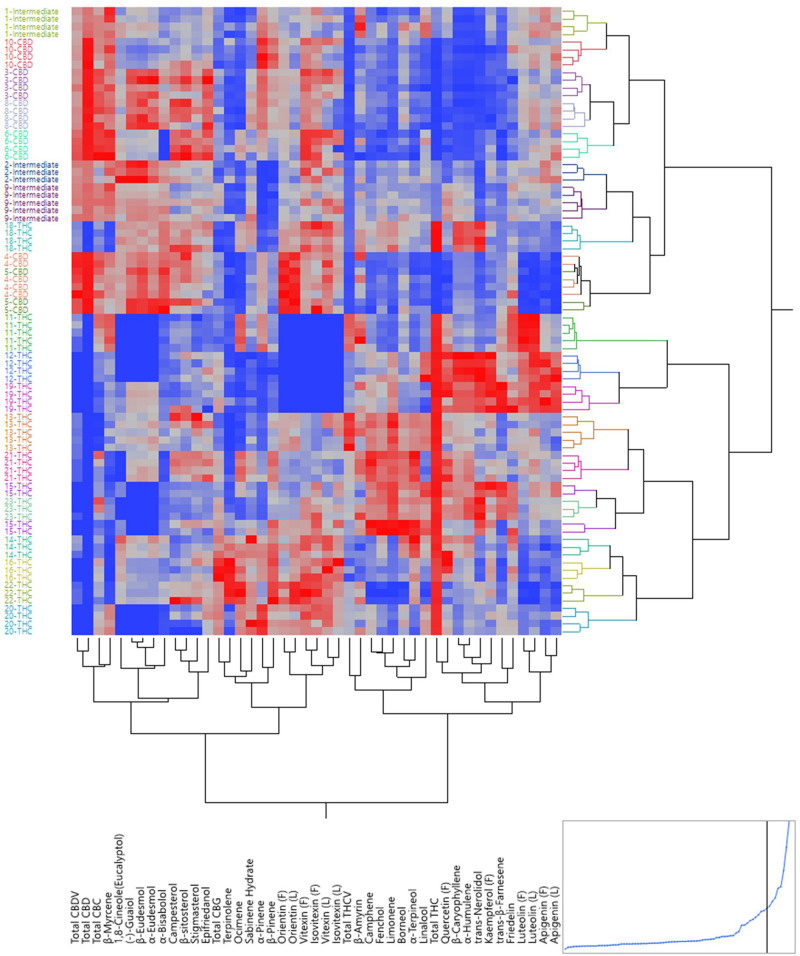
Dendrogram by hierarchical clustering analysis using the full spectrum of secondary metabolites (in ratios) of 82 plants representing 21 strains.

The same set of data was used to build a dendrogram of the 82 plants using hierarchical clustering, where almost all plants of the same strains were clustered together, except for one 5-CBD plant that was mixed with 4-CBD plants and plants of 15-THC that were mixed with 23-THC plants (Figure 3). The dendrogram shows two major branches: CBD dominant strains and intermediate strains together as one major branch, and THC dominant strains as the other. The dendrogram using absolute values of the secondary metabolites is shown in Supplementary Figure 4. These results both confirmed the minimum within-strain variation (between plants within each strain) and between-cluster variation (between strains within each chemotypes). The full spectrum of secondary metabolites without total THC and total CBD resulted in a dendrogram with the same grouping results (Supplementary Figure 5).

### Unsupervised Principal Component Analysis

**FIGURE 4:**
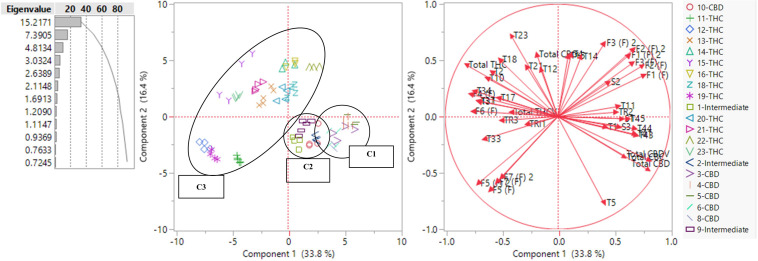
PCA scatter plot **(left)** and loading plot **(right)** using the full spectrum of secondary metabolites (in ratios) of 82 plants representing 21 strains. Terpenoids are labeled with T and the number assigned in Supplementary Table 2.5. Flavonoids are labeled as F and the number assigned in Supplementary Table 2.7. Flavonoids quantified in inflorescences are labeled (F) and flavonoids in leaf are labeled (L). Sterols are labeled as S and the number assigned in Supplementary Table 2.9. Triterpenoids are labeled as TRI and the number assigned in Supplementary Table 2.11

Figure 4 shows a scatterplot of 82 plants along two principal components (PC), where PC1 and PC2 explained 33.8 and 16.4% of the total variance, respectively. Plants of the same strains tended to occupy the same region on the plot. THC dominant strains (C3) mainly occupied the left side of the plot and CBD dominant (C1) and intermediate strains (C2) occupied the lower right quadrant. The loading matrix in Table 2 lists the compounds that contributed most to the separations along PC1 and PC2 with the absolute value of loadings equal to or greater than 0.45. PC1 was positively correlated with three cannabinoids (total CBD, total CBDV, and total CBC), one monoterpenoid (1,8-cineole (eucalyptol)), four sesquiterpenoids (β-eudesmol, (−)-guaiol, α-eudesmol, α-bisabolol), three flavonoids (orientin, vitexin, and isovitexin), three sterols (campesterol, stigmasterol, and β-sitosterol), and one triterpenoid (epifriedanol), which were compounds identified as positively correlated with total CBD. PC1 was negatively correlated with one cannabinoid (total THC), four monoterpenoids (limonene, camphene, fenchol, and linalool), four sesquiterpenoids (α-humulene, β-caryophyllene, trans-nerolidol, and trans-β-farnesene), four flavonoids (quercetin, kaempferol, and apigenin), and one triterpenoid (friedelin), which were compounds identified as positively correlated with total THC. THC dominant strains were scattered in both lower left quadrant and upper right quadrant along PC2. Compounds positively correlated with PC2 and negatively correlated with PC1 (PC1 < 0 and PC2 > 0), including total THC, total CBG, total THCV, α-terpineol, camphene, fenchol, linalool, ocimene, borneol, α-humulene, β-caryophyllene, trans-nerolidol, quercetin, and kaempferol, were more abundant in THC dominant strains than those in CBD dominant and intermediate strains. β-Myrcene was negatively correlated with PC2 and positively correlated with PC1, which means it was more abundant in CBD dominant and intermediate strains. Two flavonoids, luteolin and apigenin, were negatively correlated with PC1 and PC2, and were more abundant in THC dominant strains in the left lower quadrant than other THC dominant strains. Although some compounds were more correlated with CBD, they may be more abundant in some THC dominant strains. For example, compounds positively correlated with PC2 and positively correlated with PC1, including orientin (L), vitexin (L), and isovitexin (L), were more abundant in THC dominant strains in the upper right quadrant than strain in C1 and C2, even though these flavonoids were positively correlated with CBD. This may be the result of extensive strain crossing and hybridization. PCA using absolute values of the secondary metabolites are also shown in Supplementary Figure 6. The full spectrum of secondary metabolites without total THC and total CBD resulted in a similar PCA scatter plot where PC1 and PC2 explained 32.6 and 16.1% of the total variance, respectively (Supplementary Figure 7).

**TABLE 2:** Formatted loading matrix for PC1 and PC2 (only compounds with absolute loadings >0.45 are listed).

PC1				PC2			
	
Compound	Positive loadings	Compound	Negative loadings	Compound	Positive loadings	Compound	Negative loadings
Total CBDV	0.82	Total THC	−0.81	α-Terpineol	0.72	β-Myrcene	−0.77
Total CBD	0.81	Quercetin (F)	−0.77	Isovitexin (L)	0.65	Luteolin (F)	−0.65
Orientin (F)	0.77	Kaempferol (F)	−0.75	Vitexin (L)	0.60	Luteolin (L)	−0.60
Vitexin (F)	0.76	α-Humulene	−0.74	β-Pinene	0.56	Apigenin (F)	−0.58
β-Eudesmol	0.70	Luteolin (L)	−0.71	Total CBG	0.55	Apigenin (L)	−0.55
α-Eudesmol	0.69	trans-Nerolidol	−0.71	Orientin (L)	0.55	Total CBD	−0.47
(−)-Guaiol	0.68	β-Caryophyllene	−0.70	Terpinolene	0.54		
Vitexin (L)	0.68	trans-β-Farnesene	−0.65	Sabinene Hydrate	0.53		
Isovitexin (F)	0.67	Limonene	−0.63	Fenchol	0.50		
Orientin (L)	0.64	Luteolin (F)	−0.60	Isovitexin (F)	0.46		
α-Bisabolol	0.62	Camphene	−0.59	Vitexin (F)	0.45		
Total CBC	0.59	Apigenin (F)	−0.54	Borneol	0.45		
Campesterol	0.57	Linalool	−0.53				
β-sitosterol	0.55	Fenchol	−0.52				
1,8-Cineole (Eucalyptol)	0.54	Apigenin (L)	−0.50				
Epifriedanol	0.52	Friedelin	−0.50				
Stigmasterol	0.45						

### Supervised Canonical Correlation Analysis

**FIGURE 5:**
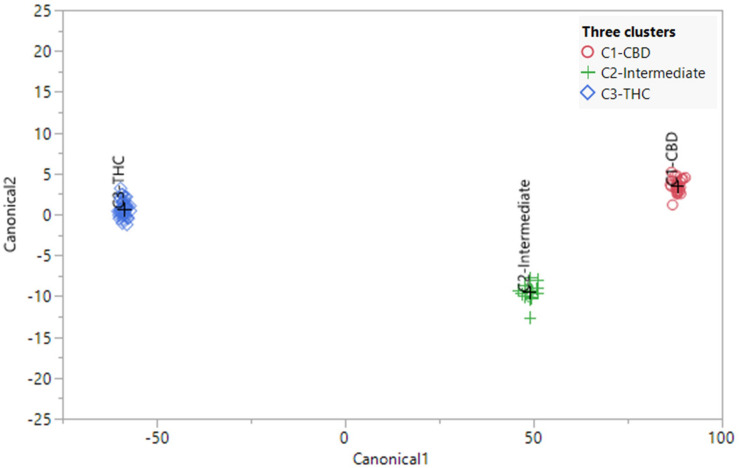
Canonical correlation analysis using the full spectrum of secondary metabolites (using ratios) of 82 plants representing 21 strains. The plants were preassigned to three chemotypes in Table 1. The observations and the multivariate means of each group (“+”) are represented as points on the biplot. An ellipse denoting a 50% contour is plotted for each group, that contains approximately 50% of the observations.

The canonical correlation analysis of 82 plants showed good separation between the three chemotypes (Figure 5). Each plant was predicted to be in its originally preassigned cluster with 100% accuracy (Supplementary Table 4). Canonical correlation analysis using the absolute values of 45 compounds were also investigated (Supplementary Figure 8), with 100% accuracy in sorting each plant into its originally preassigned chemotypes. The full spectrum of secondary metabolites, absent total THC and total CBD, also predicted each plant to be in its originally preassigned cluster with 100% accuracy (Supplementary Figure 9). However, the distance between three clusters were smaller along two canonical axes due to reduced differences in the chemical profiles of three chemotypes after removing the THC and CBD data.

### Identification of Chemotypic Markers for Three Chemotypes

**FIGURE 6:**
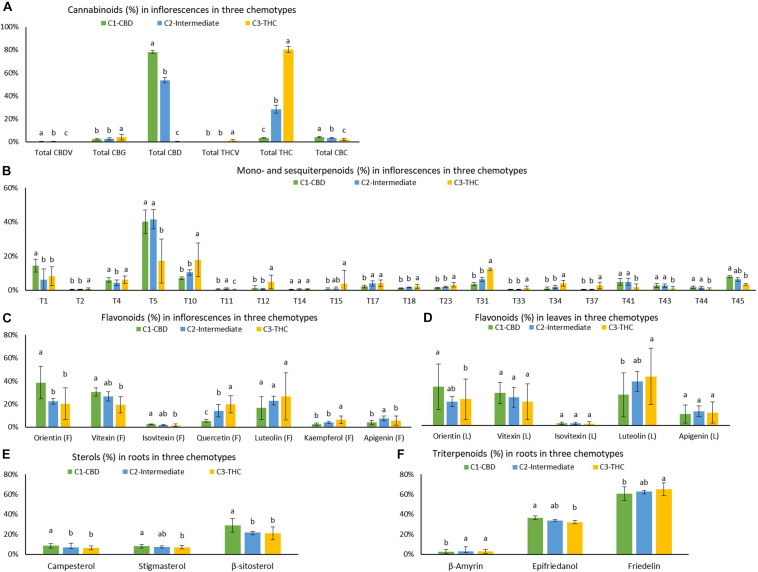
Means and standard deviations (±SD) of **(A)** cannabinoids in inflorescences, **(B)** mono- and sesquiterpenoids in inflorescences, **(C)** flavonoids in inflorescences, **(D)** flavonoids in leaves, **(E)** sterols in roots, and **(F)** triterpenoids in roots (in ratios) for each of the three chemotypes C1 – CBD dominant, C2 – intermediate, and C3 – THC dominant. Cluster means were expressed as mean ± SD. *Levels not connected by the same letter are significantly different by Tukey HSD multiple tests at the 0.05 significance level.

Means (±SD), Tukey HSD multiple tests at the 0.05 significance level, and p value of one-way ANOVA of 45 quantifiable compounds (using ratios) for each of the three chemotypes are listed in Supplementary Table 5 and plotted in Figure 6. The largest number of significant differences (Tukey HSD multiple tests at the 0.05 significance level) was 37, which was between C1 and C3. The most similar pair was C1 and C2, with 14 significant differences. The number of significant differences between C2 and C3 was 23. Strains from C1 had significant higher amount of total CBD, total CBDV, total CBC, α-pinene, β-pinene, β-myrcene, (−)-guaiol, β-eudesmol, α-eudesmol, α-bisabolol, orientin (F), vitexin (F), isovitexin (F), orientin (L), campesterol, stigmasterol, β-sitosterol, and epifriedanol than in strains of C3, which were all positively correlated with total CBD. Strains from C3 had significant higher amount of total THC, total THCV, total CBG, camphene, limonene, ocimene, linalool, fenchol, borneol, α-terpineol, β-caryophyllene, trans-β-farnesene, α-humulene, trans-nerolidol, quercetin (F), kaempferol (F), β-amyrin, and friedelin, which were all positively correlated with total THC. Most compounds in the C2 strains were at the same level with strains in C1 or C3 or at an intermediate level between C1 and C3.

Means ± SD, Tukey’s HSD multiple tests at the 0.05 significance level, and *p* value of one-way ANOVA of the absolute values of 45 compounds for each cluster were summarized in Supplementary Table 6. The largest number of significant differences was 38, which was between C1 and C3. The most similar pair was C1 and C2, with 10 differences. The number of significant differences between C2 and C3 was 23. Cannabinoids, terpenoids, flavonoids, sterols, and triterpenoids that were significantly higher in C1, C2, and C3 were similar to those identified using ratios.

Although numerous significant differences in compounds were found amongst CBD dominant, intermediate, and THC dominant strains, the group means of some compounds differed by less than a factor of two. In addition, some compounds may be significantly different qualitatively in ratios but not quantitatively in absolute values. For example, all three sterols (campesterol, stigmasterol, and β-sitosterol), were significantly higher in roots of CBD dominant strains than in THC dominant strains by ratios (one-way ANOVA *p* < 0.0001, *p* = 0.1279, and *p* < 0.0001, respectively), but they were not significantly different by absolute values (one-way ANOVA *p* = 0.1279, *p* = 0.0361, and *p* = 0.0169, respectively). Compounds significantly different (one-way ANOVA *p* < 0.05) with two or more than two-fold higher in terms of both ratios and absolute values in the identified clusters than in the clusters with the lowest values were selected as chemotypic markers. These included three cannabinoids (total CBD, total CBDV, and total CBC), six terpenoids (α-pinene, β-myrcene, (−)-guaiol, β-eudesmol, α-eudesmol, and α-bisabolol), and three flavonoids (orientin, vitexin, and isovitexin) for CBD dominant strains, three cannabinoids (total THC, total THCV, and total CBG), twelve terpenoids (camphene, limonene, ocimene, sabinene hydrate, terpinolene, linalool, fenchol, α-terpineol, β-caryophyllene, trans-β-farnesene, α-humulene, and trans-nerolidol), and two flavonoids (quercetin and kaempferol) for THC dominant strains. Intermediate strains are more similar to CBD dominant strains than THC dominant strains with higher amounts of β-myrcene, (−)-guaiol, β-eudesmol, α-eudesmol, and α-bisabolol. There are more mono- and sesquiterpenoids that are significantly higher in the THC dominant cluster than in the CBD dominant and intermediate clusters. The simultaneous presence of a collection of compounds can be used to differentiate types of plants.

## Discussion

### Cannabinoids as Chemotypic Markers

In this study, the average THC to CBD ratios in the three chemotypes were 247 ± 79, 0.5 ± 0.1, and 0.04 ± 0.01, respectively. These ratios showed that THC levels in THC dominant strains were greater than CBD levels in CBD dominant strains. This bias toward higher THC is due to the long history of extensive hybridization for recreational purposes (McPartland, 2017). A THC/CBD ratio of 247:1 in THC dominant strains matched with those in “Sativa” and “Indica” strains that were almost devoid of CBD (Fischedick et al., 2010; Hazekamp and Fischedick, 2012; Fischedick, 2015, 2017; Hazekamp et al., 2016; Jin et al., 2020). Due to CBD’s therapeutic potential without psychoactive effects (Booz, 2011; Couch et al., 2017; Vallée et al., 2017; Callejas et al., 2018; Mallada Frechín, 2018), breeding for high CBD concentrations began only recently by integrating hemp-type CBD acid synthase gene clusters into a background of drug-type cannabis to elevate CBDA production (Clarke and Merlin, 2016; Grassa et al., 2018). The CBD to THC ratios in intermediate trains were similar to 1.8:1 in our previously reported values (Jin et al., 2020), and also matched with the reported cannabinoid profile of intermediate strains available in the database. These intermediate strains may have been created by crossing purebred THC dominant types with CBD dominant types (de Meijer et al., 2003). Chemotaxonomic research in minor cannabinoids of the three chemotypes are sparse in the current literature. In this study, minor cannabinoids were mostly less than 1% in all three chemotypes and several minor cannabinoids were more abundant in one chemotypes relative to others.

### Mono- and Sesquiterpenoids as Chemotypic Markers

In general, sesquiterpenoids are considered as more stable markers because monoterpenes are more volatile (McPartland, 2017). In this study, (−)-guaiol, β-eudesmol, α-eudesmol, and α-bisabolol were identified as chemotypic markers in CBD and intermediate strains. These compounds were also noted by Hillig as signature peaks on chromatograms for pre-hybridization Afghani WLD landraces (Hillig, 2005a) and modern “Indica” dominant strains (WLD), but were present in lower amounts in pre-hybridization NLD landraces and modern “Sativa” dominant strains (NLD) (Fischedick et al., 2010; Hazekamp et al., 2016). CBD dominant strains and pre-hybridization Afghani WLD landraces are similar in that they both have elevated CBD concentrations compared to their THC dominant counterparts. According to the correlation analysis in this study, these chemotypic markers for CBD dominant strains and intermediate strains may be related to CBD production. For modern “Indica” dominant strains (WLD), which are nearly devoid of CBD, even though these sesquiterpenoids were considered to be inherited from their WLD landrace ancestors despite selection for elevated THC/CBD ratios, these compounds were detected only in trace amounts (Fischedick et al., 2010; Hazekamp and Fischedick, 2012; Fischedick, 2015, 2017; Hazekamp et al., 2016). In this study, terpinolene, β-caryophyllene, and trans-β-farnesene, were identified as chemotypic markers in THC dominant strains. These compounds were also noted by Hillig as signature peaks on chromatograms for pre-hybridization NLD landraces (Hillig, 2005a) and modern “sativa” dominant strains (NLD), but were present in lower amounts in pre-hybridization WLD landraces and modern “Indica” dominant strains (WLD) (Fischedick et al., 2010; Hazekamp et al., 2016). THC dominant strains and pre-hybridization NLD landraces both have elevated THC concentrations and are almost devoid of CBD. These chemotypic markers for THC dominant strains and intermediate strains may be correlated with THC production when CBD is not produced.

Studies have shown that terpenes in cannabis are derived from two pathways: the plastidial methylerythritol phosphate (MEP) pathway and the cytosolic mevalonate (MVA) pathway (Andre et al., 2016; Booth et al., 2017; Zager et al., 2019). Geranyl diphosphate (GPP) is typically derived from the MEP pathway and is the precursor for cannabinoid and monoterpenoid biosynthesis. Farnesyl diphosphate (FPP) is commonly produced from MVA pathway and is the precursor for sesquiterpenoids, triterpenoids and sterols. Although it is hypothesized that the identified chemotypic markers may be related to CBD or THC production, currently there are no biomedical studies on these correlations. Future studies are needed on the biochemical relationship between CBD or THC production and individual terpenoid production.

Of the strains with a reported Sativa/Hybrid/Indica ancestry label, CBD dominant strains contained two “Sativa” strains, intermediate strains contained one “Sativa” strain and one “Indica” strain, and THC dominant strains contained ten “Indica” strains and one “50/50 hybrid” strain. Based on the reported ancestry, the results of this study seem to contradict other studies. The terpenoids markers in CBD dominant strains (reported as “Sativa” due to narrow leaflets) were similar to those identified in “Indica” dominant strains but different from those identified in “Sativa” dominant strains in other studies (Fischedick et al., 2010; Hazekamp and Fischedick, 2012; Fischedick, 2015, 2017; Hazekamp et al., 2016). Similarly, the terpenoids markers in THC dominant strains (reported as “Indica” due to wide leaflets) were similar to those identified in “Sativa” dominant strains but different from those identified in “Indica” dominant strains in other studies. These conflicting results reflects the unreliability of the vernacular “Sativa” and “Indica” categories, which are based on the visual determination of leaflet shape, often with no reference data for categorization (Jin et al., 2021). This may lead to mixed results in separating modern strains genetically or chemically (Elzinga et al., 2015; Sawler et al., 2015). Another explanation for the discrepancy is that instead of separating “Sativa” vs “Indica”, which are often THC dominant strains, this paper focused on the differentiation between three chemotypes. Because no “Sativa” strains were reported for THC dominant strains in this study, whether (−)-guaiol, β-eudesmol, α-eudesmol, and α-bisabolol are more abundant in “Indica” dominant strains and terpinolene, β-caryophyllene, and trans-β-farnesene are more abundant in “Sativa” dominant strains as described in other studies could not be verified.

### Flavonoids as Chemotypic Markers

Flavonoid variation in cannabis was investigated by Clark and Bohm (1979), the only such study that used flavonoids for chemotaxonomy and for supporting a two-species hypothesis: where luteolin was more often detected in *C. sativa* L. but not in *C. indica* Lam. (Clark and Bohm, 1979). There have yet to be chemotaxonomic studies of flavonoids across the three cannabis chemotypes. We found that orientin, vitexin, and isovitexin were the signature flavonoids of CBD dominant strains, and quercetin and kaempferol were detected only in inflorescences and tended to be higher in THC dominant strains.

### Sterols and Triterpenoids as Chemotypic Markers

The role of sterols and triterpenoids in the chemotaxonomy of cannabis have not yet been investigated. In this study, CBD dominant strains had significantly higher ratios of three sterols, but they differed by less than a factor of two and may not provide a firm basis for chemotaxonomic distinction. Similarly, for triterpenoids, although the ratio of epifriedanol was higher in CBD dominant strains and friedelin was higher in THC dominant strains, the differences were not sufficiently large for these compounds to be used as chemotype markers.

### The Potential of Developing Holistic Cannabis-Based Products and Medications

Because cannabinoids are concentrated in cannabis inflorescences, cannabis leaves, stems, and roots are normally discarded by cannabis growers. However, in traditional Chinese medicine, cannabis leaves were used for treating conditions such as malaria, panting, roundworm, scorpion stings, hair loss, graying of hair. Cannabis stem bark was used for strangury and physical injury. Cannabis roots were used for gout, arthritis, joint pain, fever, skin burns, hard tumors, childbirth, and physical injury (Smith and Stuart, 1911; Brand and Wiseman, 2008; Ryz et al., 2017). Their traditional uses may serve as points of reference for investigating the medical potential of what is currently a byproduct or plant waste.

To link the traditional therapeutic uses for each part with the chemistry, we had identified the major groups of compounds in each plant part for correlation with benefits described in the literature. Cannabinoids, including THC, CBD, CBG, CBC, THCV, CBN, and CBDV, in both acid and neutral forms all have broad therapeutic potential, including anti-inflammatory (Bolognini et al., 2010; DeLong et al., 2010; De Petrocellis et al., 2012; Borrelli et al., 2013; Cascio and Pertwee, 2014; Brierley et al., 2016), analgesic (Davis and Hatoum, 1983; Evans, 1991; Cascio and Pertwee, 2014), anticonvulsant (Dwivedi and Harbison, 1975; Hill et al., 2010, 2013), antioxidant, and neuroprotective properties (Gugliandolo et al., 2018). Increasing numbers of studies have shown that minor cannabinoids significantly contribute to the variance among cannabis extract, which further alter or enhance targeted therapeutic effects comparing to pure THC or CBD alone (Berman et al., 2018; Baram et al., 2019).

Terpenoids are widely distributed in highly fragrant fruits, plants, and herbs and they have anti-inflammatory (Miguel, 2010; Xiao et al., 2018), antirheumatic (Ames-Sibin et al., 2018), pain relieving (Gouveia et al., 2018; Xiao et al., 2018), antioxidant and neuroprotective (Shahriari et al., 2018), gastroprotective (Tambe et al., 1996; Klopell et al., 2007), and larvicidal properties (Govindarajan et al., 2016). If a cannabinoid-terpenoid entourage effect exists, it may not be at the CB1 or CB2 receptor level, but rather the terpenoids may act at different molecular targets in neuronal circuits (Santiago et al., 2019).

Flavonoids share a wide range of biological effects with cannabinoids and terpenoids, including anti-inflammatory (He et al., 2016; Hayasaka et al., 2018; Sharma et al., 2018; Wang et al., 2018; Yao et al., 2018), antirheumatic (Haleagrahara et al., 2017; Chirumbolo and Bjørklund, 2018; Yang et al., 2018), analgesic (He et al., 2016; Strada et al., 2017), and antioxidant and neuroprotective properties (An et al., 2012; He et al., 2016; Ashaari et al., 2018; Tsai et al., 2018; Wang et al., 2018; Zheng et al., 2018). Ginkgo leaves are one of the prominent sources of flavonoids, with 0.4% total flavonoids in terms of total aglycones (Hasler et al., 1992). In this study, the mean of total flavonoids was 0.19 ± 0.09%, which makes cannabis leaves a promising source for flavonoids extraction.

Sterols and triterpenoids are mainly present in cannabis stem bark and roots. Friedelin is the most abundant and most studied triterpenoids in cannabis, and has anti-inflammatory, antioxidant, estrogenic, anti-cancer, and liver protectant properties (Ryz et al., 2017). β-sitosterol, stigmasterol, and campesterol are the most abundant phytosterols in the human diet. Phytosterols are widely recognized as lowering the levels of low-density lipoprotein cholesterol (Gylling et al., 2014; Ras et al., 2014). They are also studied for anti-inflammatory, antioxidant, and pain relieving properties (Kozłowska et al., 2016).

These groups of identified bioactive compounds may underpin the traditional applications indicated for each plant part, but most of the therapeutic properties for these individual compounds have been studied in other herbal medicine and not in cannabis. The pharmaceutical values and the potential synergies of these bioactive compounds need to be directly investigated using cannabis material. Well-designed clinical studies are necessary to convert each part of the cannabis plant into evidence-based medicine. The chemotypic markers identified in this study will facilitate strain selection in research and clinical studies when the optimal combination of the chemical compounds is determined for treating certain conditions.

## Conclusion

The chemical variation in CBD dominant and intermediate strains has yet to be studied or compared to THC dominant strains in the literature. This comprehensive chemotaxonomic investigation profiled cannabinoids, terpenoids, flavonoids, sterols, and triterpenoids in inflorescences, leaves, stem bark, and roots in 82 plants of 21 cannabis strains. These chemical data were subjected to correlation analysis, unsupervised clustering analysis (hierarchical clustering and PCA) and supervised canonical correlations analysis. In unsupervised clustering, 82 plants were clustered in accordance with their chemotypes. Canonical correlation analysis classified 82 plants into three chemotypes with 100% accuracy using full spectrum of secondary metabolites. Numerous significant differences that could be used as chemotypic markers were found amongst CBD dominant, intermediate, and THC dominant strains. These identified compounds were largely consistent with results from correlation analysis, hierarchical clustering, PCA, and by comparing concentration and ratio averages between chemotypes. At each step of the clustering analysis, it was found that secondary metabolites without total THC and total CBD could continue to sort strains into their defined chemotypes and achieve the same clustering results. This demonstrated that the clustering results were not solely driven by THC and CBD content or ratio, and that other metabolites can be used as chemotypic markers. However, the robustness of these markers should be tested in different growing environments to truly elucidate the chemical differences in terms of chemotypes or intra-chemotype sub-clusters. The results of this study provide a proof-of-concept for further collaboration between academia and the industry for leveraging chemotypic markers in medical studies and clinical trials.

## Data Availability Statement

The original contributions presented in the study are included in the article/Supplementary Material, further inquiries can be directed to the corresponding author.

## Author Contributions

DJ conceived the project, designed the experiments, preformed the experiments, collected and analyzed the data, and wrote the manuscript. PH contacted the licensed cultivator for this project and proofread the manuscript. JS provided funding, provided suggestions, and proofread the manuscript. JC was the supervisory author and monitored the research progress, provided suggestions, and finalized the manuscript. All authors contributed to the article and approved the submitted version.

## Conflict of Interest

DJ and JS were employed by the company PBG BioPharma Inc. PH was employed by the company Egret Bioscience and Lighthouse Genomics. The remaining authors declare that the research was conducted in the absence of any commercial or financial relationships that could be construed as a potential conflict of interest.

## Footnotes

1 https://www.leafly.ca/

## Supplementary Material

Supplementary Figure 1 Secondary metabolites profiling in cannabis roots, stem bark, leaves, and inflorescences in 82 plants of 21 strains.

Supplementary Figure 2 Correlations of total THC and total CBD with terpenoids using content ratios (%/%) in (A) inflorescences and in (B) leaves.

Supplementary Figure 3 Correlations of total THC and total CBD with cannabinoids (in inflorescences), mono- and sesquiterpenoids (in inflorescences), flavonoids (in inflorescences and leaves), sterols and triterpenoids (in roots) on quantifiable compounds using ratios.

Supplementary Figure 4 Dendrogram by hierarchical clustering analysis using the full spectrum of secondary metabolites (absolute values) of 82 plants representing 21 strains.

Supplementary Figure 5 Dendrogram by hierarchical clustering analysis using the full spectrum of secondary metabolites (using ratios) without total THC and total CBD.

Supplementary Figure 6 PCA scatter plot (left) and loading plot (right) using the full spectrum of secondary metabolites (absolute values) of 82 plants representing 21 strains.

Supplementary Figure 7 PCA scatter plot (left) and loading plot (right) using the full spectrum of secondary metabolites (using ratios) without total THC and total CBD.

Supplementary Figure 8 Canonical correlation analysis using the full spectrum of secondary metabolites (absolute values) of 82 plants representing 21 strains.

Supplementary Figure 9 Canonical correlation analysis using the full spectrum of secondary metabolites (using ratios) of 82 plants representing 21 strains.

Supplementary Table 1 Strain information and assignment of 21 strains into three chemotypes.

Supplementary Table 2.1 Secondary metabolites profiled in inflorescences of 82 plants of 21 strains.

Supplementary Table 2.2 Cannabinoids profiled in inflorescences of 82 plants for three chemotypes.

Supplementary Table 2.3 Cannabinoid profile in leaves of 82 plants for three chemotypes.

Supplementary Table 2.4 Cannabinoid profile in stem bark of 82 plants for three chemotypes.

Supplementary Table 2.5 Mono- and sesquiterpenoids profile in inflorescences of 82 plants for three chemotypes.

Supplementary Table 2.6 Mono- and sesquiterpenoids profile in leaves of 82 plants for three chemotypes.

Supplementary Table 2.7 Flavonoids profile in inflorescences for three chemotypes.

Supplementary Table 2.8 Flavonoids profile in leaves for three chemotypes.

Supplementary Table 2.9 Sterols profile in stem bark for three chemotypes.

Supplementary Table 2.10 Sterols profile in roots for three chemotypes.

Supplementary Table 2.11 Triterpenoids profile in stem bark for three chemotypes.

Supplementary Table 2.12 Triterpenoids profile in roots for three chemotypes.

Supplementary Table 3 Correlations of total THC and total CBD with minor cannabinoids, mono- and sesquiterpenoids, flavonoids, sterols, and triterpenoids (only positive correlations are shown).

Supplementary Table 4 Summary prediction of 82 plants into preassigned chemotypes using Canonical correlation analysis (using ratios).

Supplementary Table 5 Means (±SD) of the ratios of 45 secondary metabolites above quantification limit for 82 plants assigned to C1-CBD dominant, C2-intermediate, and C3-THC dominant.

Supplementary Table 6 Means (±SD) of the absolute values of 45 secondary metabolites (mg/mg%) for 82 plants assigned to C1-CBD dominant, C2-intermediate, and C3-THC dominant.

Supplementary Table 7 Secondary metabolites used in correlation analysis and classification analysis.

Supplementary Table 8 Correlations of cannabinoids and terpenoids in inflorescences and leaves.

Supplementary Table 9 Secondary metabolites in all plant parts (absolute values).

## References

[B1] Aizpurua-Olaizola O. S. (2016). Evolution of the cannabinoid and terpene content during the growth of *Cannabis sativa* plants from different chemotypes. *J. Nat. Prod.* 79 324–331. 10.1021/acs.jnatprod.5b00949 26836472

[B2] Ames-Sibin A. P. Barizão C. L. Castro-Ghizoni C. V. Silva F. M. S. Sá-Nakanishi A. B. Bracht L. (2018). β-Caryophyllene, the major constituent of copaiba oil, reduces systemic inflammation and oxidative stress in arthritic rats. *J. Cell. Biochem.* 119 10262–10277. 10.1002/jcb.27369 30132972

[B3] An F. Yang G. Tian J. Wang S. (2012). Antioxidant effects of the orientin and vitexin in *Trollius chinensis* Bunge in D-galactose-aged mice. *Neural Regen. Res.* 7:2565. 10.3969/j.issn.1673-5374.2012.33.001 PMC4200723 25368632

[B4] Andre C. M. Hausman J.-F. Guerriero G. (2016). *Cannabis sativa*: the plant of the thousand and one molecules. *Front. Plant Sci.* 7:19. 10.3389/fpls.2016.00019 26870049 PMC4740396

[B5] Ashaari Z. Hassanzadeh G. Alizamir T. Yousefi B. Keshavarzi Z. Mokhtari T. (2018). The flavone luteolin improves central nervous system disorders by different mechanisms: a review. *J. Mol. Neurosci.* 65 491–506. 10.1007/s12031-018-1094-2 30083786

[B6] Avraham Y. Grigoriadis N. C. Poutahidis T. Vorobiev’ L. Magen I. Ilan Y. (2011). Cannabidiol improves brain and liver function in a fulminant hepatic failure-induced model of hepatic encephalopathy in mice. *Br. J. Pharmacol.* 162 1650–1658. 10.1111/j.1476-5381.2010.01179.x 21182490 PMC3057300

[B7] Baram L. Peled E. Berman P. Yellin B. Besser E. Benami M. (2019). The heterogeneity and complexity of *Cannabis* extracts as antitumor agents. *Oncotarget* 10 4091–4106. 10.18632/oncotarget.26983 31289609 PMC6609248

[B8] Berman P. Futoran K. Lewitus G. M. Mukha D. Benami M. Shlomi T. (2018). A new ESI-LC/MS approach for comprehensive metabolic profiling of phytocannabinoids in *Cannabis*. *Sci. Rep.* 8 1–15. 30250104 10.1038/s41598-018-32651-4 PMC6155167

[B9] Blasco-Benito S. Seijo-Vila M. Caro-Villalobos M. Tundidor I. Andradas C. García-Taboada E. (2018). Appraising the “entourage effect”: antitumor action of a pure cannabinoid versus a botanical drug preparation in preclinical models of breast cancer. *Biochem. Pharmacol.* 157 285–293. 10.1016/j.bcp.2018.06.025 29940172

[B10] Bloomfield M. A. P. Green S. F. Hindocha C. Yamamori Y. Yim J. L. L. Jones A. P. M. (2020). The effects of acute cannabidiol on cerebral blood flow and its relationship to memory: an arterial spin labelling magnetic resonance imaging study. *J. Psychopharmacol. Oxf. Engl.* 34 981–989. 10.1177/0269881120936419 32762272 PMC7436497

[B11] Bolognini D. Costa B. Maione S. Comelli F. Marini P. Di Marzo V. (2010). The plant cannabinoid Δ9-tetrahydrocannabivarin can decrease signs of inflammation and inflammatory pain in mice. *Br. J. Pharmacol.* 160 677–687. 10.1111/j.1476-5381.2010.00756.x 20590571 PMC2931567

[B12] Booth J. K. Page J. E. Bohlmann J. (2017). Terpene synthases from *Cannabis sativa*. *PLoS One* 12:e0173911. 10.1371/journal.pone.0173911 28355238 PMC5371325

[B13] Booz G. W. (2011). Cannabidiol as an emergent therapeutic strategy for lessening the impact of inflammation on oxidative stress. *Free Radic. Biol. Med.* 51 1054–1061. 10.1016/j.freeradbiomed.2011.01.007 21238581 PMC3085542

[B14] Borrelli F. Fasolino I. Romano B. Capasso R. Maiello F. Coppola D. (2013). Beneficial effect of the non-psychotropic plant cannabinoid cannabigerol on experimental inflammatory bowel disease. *Biochem. Pharmacol.* 85 1306–1316. 10.1016/j.bcp.2013.01.017 23415610

[B15] Brand E. Wiseman N. (2008). *Concise Chinese Materia Medica.* Taos, NM: Paradigm Publications.

[B16] Brand E. J. Zhao Z. (2017). *Cannabis* in chinese medicine: are some traditional indications referenced in ancient literature related to cannabinoids? *Front. Pharmacol.* 8:108. 10.3389/fphar.2017.00108 28344554 PMC5345167

[B17] Brierley D. I. Samuels J. Duncan M. Whalley B. J. Williams C. M. (2016). Cannabigerol is a novel, well-tolerated appetite stimulant in pre-satiated rats. *Psychopharmacology* 233 3603–3613. 10.1007/s00213-016-4397-4 27503475 PMC5021742

[B18] Callejas G. H. Figueira R. L. Gonçalves F. L. L. Volpe F. A. P. Zuardi A. W. Crippa J. A. (2018). Maternal administration of cannabidiol promotes an anti-inflammatory effect on the intestinal wall in a gastroschisis rat model. *Br. J. Med. Biol. Res.* 51:e7132. 10.1590/1414-431X20177132 PMC5875904 29561958

[B19] Caplan D. M. (2018). *Propagation and Root Zone Management for Controlled Environment Cannabis Production.* Ph.D. Thesis. Guelph, ON: University of Guelph.

[B20] Cascio M. G. Pertwee R. G. (2014). “Known pharmacological actions of nine nonpsychotropic phytocannabinoids,” in *Handbook of Cannabis*, ed. Pertwee R. G. (Oxford: Oxford University Press).

[B21] Chirumbolo S. Bjørklund G. (2018). Quercetin in collagen-induced arthritis. Some comments. *Int. Immunopharmacol.* 62:335. 10.1016/j.intimp.2018.06.003 29970297

[B22] Clark M. N. Bohm B. A. (1979). Flavonoid variation in *Cannabis* L. *Bot. J. Linn. Soc.* 79 249–257. 10.1111/j.1095-8339.1979.tb01517.x

[B23] Clarke R. C. Merlin M. D. (2016). *Cannabis* domestication, breeding history, present-day genetic diversity, and future prospects. *Crit. Rev. Plant Sci.* 35 293–327. 10.1080/07352689.2016.1267498

[B24] Couch D. G. Tasker C. Theophilidou E. Lund J. N. O’Sullivan S. E. (2017). Cannabidiol and palmitoylethanolamide are anti-inflammatory in the acutely inflamed human colon. *Clin. Sci.* 131 2611–2626. 10.1042/cs20171288 28954820

[B25] Davis W. M. Hatoum N. S. (1983). Neurobehavioral actions of cannabichromene and interactions with delta 9-tetrahydrocannabinol. *Gen. Pharmacol.* 14 247–252. 10.1016/0306-3623(83)90004-6 6301931

[B26] de Meijer E. P. M. Bagatta M. Carboni A. Crucitti P. Moliterni V. M. C. Ranalli P. (2003). The inheritance of chemical phenotype in *Cannabis sativa* L. *Genetics* 163 335–346. 10.1093/genetics/163.1.335 12586720 PMC1462421

[B27] de Meijer E. P. M. Hammond K. M. Sutton A. (2009). The inheritance of chemical phenotype in *Cannabis sativa* L. (IV): cannabinoid-free plants. *Euphytica* 168 95–112. 10.1007/s10681-009-9894-7

[B28] De Petrocellis L. Orlando P. Moriello A. S. Aviello G. Stott C. Izzo A. A. (2012). Cannabinoid actions at TRPV channels: effects on TRPV3 and TRPV4 and their potential relevance to gastrointestinal inflammation. *Acta Physiol.* 204 255–266. 10.1111/j.1748-1716.2011.02338.x 21726418

[B29] DeLong G. T. Wolf C. E. Poklis A. Lichtman A. H. (2010). Pharmacological evaluation of the natural constituent of *Cannabis sativa*, cannabichromene and its modulation by Δ9-tetrahydrocannabinol. *Drug Alcohol Depend.* 112 126–133. 10.1016/j.drugalcdep.2010.05.019 20619971 PMC2967639

[B30] Dwivedi C. Harbison R. D. (1975). Anticonvulsant activities of Δ-8 and Δ-9 tetrahydrocanabinol and uridine. *Toxicol. Appl. Pharmacol.* 31 452–458. 10.1016/0041-008x(75)90268-9 1145630

[B31] ElSohly M. A. Gul W. (2014). “Constituents of *Cannabis sativa*,” in *Handbook of Cannabis*, ed. Pertwee R. G. (Oxford: Oxford University Press), 3–22. 10.1093/acprof:oso/9780199662685.003.0001

[B32] ElSohly M. A. Slade D. (2005). Chemical constituents of marijuana: the complex mixture of natural cannabinoids. *Life Sci.* 78 539–548. 10.1016/j.lfs.2005.09.011 16199061

[B33] Elzinga S. Fischedick J. Podkolinski R. Raber J. C. (2015). Cannabinoids and terpenes as chemotaxonomic markers in *Cannabis*. *Nat. Prod. Chem. Res.* 3:4.

[B34] Evans F. J. (1991). Cannabinoids: the separation of central from peripheral effects on a structural basis. *Planta Med.* 57 S60–S67. 1659702

[B35] Fischedick J. (2015). Cannabinoids and terpenes as chemotaxonomic markers in *Cannabis*. *Nat. Prod. Chem. Res.* 03:4.

[B36] Fischedick J. T. (2017). Identification of terpenoid chemotypes among high (−)-trans-Δ9-tetrahydrocannabinol-producing *Cannabis sativa* L. cultivars. *Cannabis Cannabinoid Res.* 2 34–47. 10.1089/can.2016.0040 28861503 PMC5436332

[B37] Fischedick J. T. Hazekamp A. Erkelens T. Choi Y. H. Verpoorte R. (2010). Metabolic fingerprinting of *Cannabis sativa* L., cannabinoids and terpenoids for chemotaxonomic and drug standardization purposes. *Phytochemistry* 71 2058–2073. 10.1016/j.phytochem.2010.10.001 21040939

[B38] Flores-Sanchez I. J. Verpoorte R. (2008). PKS activities and biosynthesis of cannabinoids and flavonoids in *Cannabis sativa* L. *Plants Plant Cell Physiol.* 49 1767–1782. 10.1093/pcp/pcn150 18854334

[B39] French J. Thiele E. Mazurkiewicz-Beldzinska M. Benbadis S. Marsh E. Joshi C. (2017). Cannabidiol (CBD) significantly reduces drop seizure frequency in lennox-gastaut syndrome (LGS): results of a multi-center, randomized, double-blind, placebo controlled trial (GWPCARE4)(S21.001). *Neurology* 88:S21.

[B40] Gouveia D. N. Costa J. S. Oliveira M. A. Rabelo T. K. de e Silva A. M. O. Carvalho A. A. (2018). α-Terpineol reduces cancer pain via modulation of oxidative stress and inhibition of iNOS. *Biomed. Pharmacother.* 105 652–661. 10.1016/j.biopha.2018.06.027 29902764

[B41] Govindarajan M. Rajeswary M. Hoti S. L. Bhattacharyya A. Benelli G. (2016). Eugenol, α-pinene and β-caryophyllene from *Plectranthus barbatus* essential oil as eco-friendly larvicides against malaria, dengue and Japanese encephalitis mosquito vectors. *Parasitol. Res.* 115 807–815. 10.1007/s00436-015-4809-0 26518773

[B42] Grassa C. J. Wenger J. P. Dabney C. Poplawski S. G. Motley S. T. Michael T. P. (2018). A complete *Cannabis* chromosome assembly and adaptive admixture for elevated cannabidiol (CBD) content. *bioRxiv* [Preprint]. 10.1101/458083v3

[B43] Gugliandolo A. Pollastro F. Grassi G. Bramanti P. Mazzon E. (2018). In vitro model of neuroinflammation: efficacy of cannabigerol, a non-psychoactive cannabinoid. *Int. J. Mol. Sci.* 19:1992. 10.3390/ijms19071992 29986533 PMC6073490

[B44] Gylling H. Plat J. Turley S. Ginsberg H. N. Ellegård L. Jessup W. (2014). Plant sterols and plant stanols in the management of dyslipidaemia and prevention of cardiovascular disease. *Atherosclerosis* 232 346–360. 10.1016/j.atherosclerosis.2013.11.043 24468148

[B45] Haleagrahara N. Miranda-Hernandez S. Alim M. A. Hayes L. Bird G. Ketheesan N. (2017). Therapeutic effect of quercetin in collagen-induced arthritis. *Biomed. Pharmacother.* 90 38–46. 10.1016/j.biopha.2017.03.026 28342364

[B46] Hasler A. Sticher O. Meier B. (1992). Identification and determination of the flavonoids from *Ginkgo biloba* by high-performance liquid chromatography. *J. Chromatogr. A* 605 41–48. 10.1016/0021-9673(92)85026-p

[B47] Hayasaka N. Shimizu N. Komoda T. Mohri S. Tsushida T. Eitsuka T. (2018). Absorption and metabolism of luteolin in rats and humans in relation to in vitro anti-inflammatory effects. *J. Agric. Food Chem.* 66 11320–11329. 10.1021/acs.jafc.8b03273 30280574

[B48] Hazekamp A. Fischedick J. T. (2012). *Cannabis* - from cultivar to chemovar. *Drug Test. Anal.* 4 660–667. 10.1002/dta.407 22362625

[B49] Hazekamp A. Tejkalová K. Papadimitriou S. (2016). *Cannabis*: from cultivar to chemovar II—a metabolomics approach to *Cannabis* classification. *Cannabis Cannabinoid Res.* 1 202–215. 10.1089/can.2016.0017

[B50] He M. Min J.-W. Kong W.-L. He X.-H. Li J.-X. Peng B.-W. (2016). A review on the pharmacological effects of vitexin and isovitexin. *Fitoterapia* 115 74–85. 10.1016/j.fitote.2016.09.011 27693342

[B51] Hill A. J. Weston S. E. Jones N. A. Smith I. Bevan S. A. Williamson E. M. (2010). Δ9-Tetrahydrocannabivarin suppresses in vitro epileptiform and in vivo seizure activity in adult rats. *Epilepsia* 51 1522–1532. 10.1111/j.1528-1167.2010.02523.x 20196794

[B52] Hill T. D. M. Cascio M.-G. Romano B. Duncan M. Pertwee R. G. Williams C. M. (2013). Cannabidivarin-rich *Cannabis* extracts are anticonvulsant in mouse and rat via a CB1 receptor-independent mechanism. *Br. J. Pharmacol.* 170 679–692. 10.1111/bph.12321 23902406 PMC3792005

[B53] Hillig K. W. (2005a). *A Systematic Investigation of Cannabis.* Ph.D. thesis. Bloomington, IN: Indiana University.

[B54] Hillig K. W. (2005b). Genetic evidence for speciation in *Cannabis* (Cannabaceae). *Genet. Resour. Crop Evol.* 52 161–180. 10.1007/s10722-003-4452-y

[B55] Hotelling H. (1936). Relations between two sets of variates. *Biometrika* 28 321–377. 10.2307/2333955

[B56] Jikomes N. Zoorob M. (2018). The cannabinoid content of legal *Cannabis* in Washington state varies systematically across testing facilities and popular consumer products. *Sci. Rep.* 8:4519. 10.1038/s41598-018-22755-2 PMC5852027 29540728

[B57] Jin D. Dai K. Xie Z. Chen J. (2020). Secondary metabolites profiled in *Cannabis* inflorescences, leaves, stem barks, and roots for medicinal purposes. *Sci. Rep.* 10:3309. 10.1038/s41598-020-60172-6 PMC7039888 32094454

[B58] Jin D. Henry P. Shan J. Chen J. (2021). Identification of phenotypic characteristics in three chemotype categories in the genus *Cannabis*. *HortScience* 1 1–10.

[B59] Jin D. Jin S. Chen J. (2019). *Cannabis* indoor growing conditions, management practices, and post-harvest treatment: a review. *Am. J. Plant Sci.* 10 925–946. 10.4236/ajps.2019.106067

[B60] Jin D. Jin S. Yu Y. Lee C. Chen J. (2017). Classification of *Cannabis* cultivars marketed in canada for medical purposes by quantification of cannabinoids and terpenes using HPLC-DAD and GC-MS. *J. Anal. Bioanal. Tech.* 8:2.

[B61] Jolliffe I. T. (2002). *Principal Component Analysis, 2nd ed, Springer Series in Statistics.* New York: Springer-Verlag.

[B62] Klopell F. C. Lemos M. Sousa J. P. B. Comunello E. Maistro E. L. Bastos J. K. (2007). Nerolidol, an antiulcer constituent from the essential oil of *Baccharis dracunculifolia* DC (Asteraceae). *Z. Naturforschung C J. Biosci.* 62 537–542. 10.1515/znc-2007-7-812 17913068

[B63] Kozłowska M. Gruczńska E. Ścibisz I. Rudzińska M. (2016). Fatty acids and sterols composition, and antioxidant activity of oils extracted from plant seeds. *Food Chem.* 213 450–456. 10.1016/j.foodchem.2016.06.102 27451203

[B64] Lynch R. C. Vergara D. Tittes S. White K. Schwartz C. J. Gibbs M. J. (2016). Genomic and chemical diversity in *Cannabis*. *Crit. Rev. Plant Sci.* 35 349–363.

[B65] Mallada Frechín J. (2018). Effect of tetrahydrocannabinol: cannabidiol oromucosal spray on activities of daily living in multiple sclerosis patients with resistant spasticity: a retrospective, observational study. *Neurodegener. Dis. Manag.* 8 151–159. 10.2217/nmt-2017-0055 29851356

[B66] Mandolino G. Bagatta M. Carboni A. Ranalli P. de Meijer E. (2003). Qualitative and quantitative aspects of the inheritance of chemical phenotype in *Cannabis*. *J. Ind. Hemp.* 8 51–72. 10.1300/j237v08n02_04 PMC1462421 12586720

[B67] McGuire P. Robson P. Cubala W. J. Vasile D. Morrison P. D. Barron R. (2018). Cannabidiol (CBD) as an adjunctive therapy in schizophrenia: a multicenter randomized controlled trial. *Am. J. Psychiatry* 175 225–231. 10.1176/appi.ajp.2017.17030325 29241357

[B68] McPartland J. M. (2017). “*Cannabis sativa* and *Cannabis indica* versus “Sativa” and “Indica.”,” in *Cannabis Sativa L.-Botany and Biotechnology*, eds Chandra S. Lata H. ElSohly M. A. (Berlin: Springer), 101–121. 10.1007/978-3-319-54564-6_4

[B69] McPartland J. M. Guy G. W. (2017). Models of ‘*Cannabis* taxonomy, cultural bias, and conflicts between scientific and vernacular names. *Bot. Rev.* 4 327–381. 10.1007/s12229-017-9187-0

[B70] McPartland J. M. Russo E. B. (2001). *Cannabis* and *Cannabis* extracts: greater than the sum of their parts? *J. Cannabis Ther.* 1 103–132. 10.1300/j175v01n03_08

[B71] Miguel M. G. (2010). Antioxidant and anti-inflammatory activities of essential oils: a short review. *Molecules* 15 9252–9287. 10.3390/molecules15129252 21160452 PMC6259136

[B72] Pollastro F. Minassi A. Fresu L. G. (2018). *Cannabis* phenolics and their bioactivities. *Curr. Med. Chem.* 25 1160–1185. 10.2174/0929867324666170810164636 28799497

[B73] Potter D. J. (2009). *The propagation, Characterisation and Optimisation of Cannabis sativa L. as a Phytopharmaceutical.* Ph.D. Thesis. London: King’s College.

[B74] Ras R. T. Geleijnse J. M. Trautwein E. A. (2014). LDL-cholesterol-lowering effect of plant sterols and stanols across different dose ranges: a meta-analysis of randomised controlled studies. *Br. J. Nutr.* 112 214–219. 10.1017/s0007114514000750 24780090 PMC4071994

[B75] Richins R. D. Rodriguez-Uribe L. Lowe K. Ferral R. O’Connell M. A. (2018). Accumulation of bioactive metabolites in cultivated medical *Cannabis*. *PLoS One* 13:e0201119. 10.1371/journal.pone.0201119 30036388 PMC6056047

[B76] Ross S. A. ElSohly M. A. Sultana G. N. Mehmedic Z. Hossain C. F. Chandra S. (2005). Flavonoid glycosides and cannabinoids from the pollen of *Cannabis sativa* L. *Phytochem. Anal. Int. J. Plant Chem. Biochem. Tech.* 16 45–48. 10.1002/pca.809 15688956

[B77] Russo E. B. (2011). Taming THC: potential *Cannabis* synergy and phytocannabinoid-terpenoid entourage effects. *Br. J. Pharmacol.* 163 1344–1364. 10.1111/j.1476-5381.2011.01238.x 21749363 PMC3165946

[B78] Russo E. B. Marcu J. (2017). *Cannabis* pharmacology: the usual suspects and a few promising leads. *Cannabinoid. Pharmacol.* 80 67–134. 10.1016/bs.apha.2017.03.004 28826544

[B79] Ryz N. R. Remillard D. J. Russo E. B. (2017). *Cannabis* roots: a traditional therapy with future potential for treating inflammation and pain. *Cannabis Cannabinoid Res.* 2 210–216. 10.1089/can.2017.0028 29082318 PMC5628559

[B80] Santiago M. Sachdev S. Arnold J. C. McGregor I. S. Connor M. (2019). Absence of entourage: terpenoids commonly found in *Cannabis sativa* do not modulate the functional activity of Δ9-THC at human CB1 and CB2 receptors. *Cannabis Cannabinoid Res.* 4 165–176. 10.1089/can.2019.0016 31559333 PMC6757242

[B81] Sawler J. Stout J. M. Gardner K. M. Hudson D. Vidmar J. Butler L. (2015). The genetic structure of marijuana and hemp. *PLoS One* 10:e0133292. 10.1371/journal.pone.0133292 26308334 PMC4550350

[B82] Shahriari M. Zibaee A. Sahebzadeh N. Shamakhi L. (2018). Effects of α-pinene, trans-anethole, and thymol as the essential oil constituents on antioxidant system and acetylcholine esterase of *Ephestia kuehniella* Zeller (Lepidoptera: Pyralidae). *Pestic. Biochem. Physiol.* 150 40–47. 10.1016/j.pestbp.2018.06.015 30195386

[B83] Sharma A. Kashyap D. Sak K. Tuli H. S. Sharma A. K. (2018). Therapeutic charm of quercetin and its derivatives: a review of research and patents. *Pharm. Pat. Anal.* 7 15–32. 10.4155/ppa-2017-0030 29227203

[B84] Small E. Beckstead H. D. (1973). Letter: cannabinoid phenotypes in *Cannabis sativa*. *Nature* 245 147–148. 10.1038/245147a0 4582664

[B85] Smith F. P. Stuart G. A. (1911). *Chinese Materia Medica: Vegetable Kingdom.* Shanghai: American Presbyterian Mission Press.

[B86] Strada C. L. Lima K. da C. da Silva V. C. Ribeiro R. V. Dores E. F. (2017). Isovitexin as marker and bioactive compound in the antinociceptive activity of the Brazilian crude drug extracts of *Echinodorus scaber* and *E. grandiflorus*. *Rev. Bras. Farmacogn.* 27 619–626. 10.1016/j.bjp.2017.05.011

[B87] Tambe Y. Tsujiuchi H. Honda G. Ikeshiro Y. Tanaka S. (1996). Gastric cytoprotection of the non-steroidal anti-inflammatory sesquiterpene, beta-caryophyllene. *Planta Med.* 62 469–470. 10.1055/s-2006-957942 9005452

[B88] Tsai M.-S. Wang Y.-H. Lai Y.-Y. Tsou H.-K. Liou G.-G. Ko J.-L. (2018). Kaempferol protects against propacetamol-induced acute liver injury through CYP2E1 inactivation, UGT1A1 activation, and attenuation of oxidative stress, inflammation and apoptosis in mice. *Toxicol. Lett.* 290 97–109. 10.1016/j.toxlet.2018.03.024 29574133

[B89] Turner C. E. Elsohly M. A. Boeren E. G. (1980). Constituents of *Cannabis sativa* L. XVII. A review of the natural constituents. *J. Nat. Prod.* 43 169–234. 10.1021/np50008a001 6991645

[B90] Turner C. E. Elsohly M. A. Cheng P. C. Lewis G. (1979). Constituents of *Cannabis sativa* L., XIV: intrinsic problems in classifying *Cannabis* based on a single cannabinoid analysis. *J. Nat. Prod.* 42 317–319. 10.1021/np50003a017

[B91] Upton R. Craker L. ElSohly M. Romm A. Russo E. Sexton M. (2014). *Cannabis Inflorescence: Cannabis spp.; Standards of Identity, Analysis, and Quality Control.* Scotts Valley, CA: American Herbal Pharmacopoeia.

[B92] Vallée A. Lecarpentier Y. Guillevin R. Vallée J.-N. (2017). Effects of cannabidiol interactions with Wnt/β-catenin pathway and PPARγ on oxidative stress and neuroinflammation in Alzheimer’s disease. *Acta Biochim. Biophys. Sin.* 49 853–866. 10.1093/abbs/gmx073 28981597

[B93] Vanhoenacker G. Van Rompaey P. De Keukeleire D. Sandra P. (2002). Chemotaxonomic features associated with flavonoids of cannabinoid-free *Cannabis* (*Cannabis sativa* subsp. sativa L.) in relation to hops (*Humulus lupulus* L.). *Nat. Prod. Lett.* 16 57–63. 10.1080/1057563029001/4863 11942684

[B94] Wang J. Li T. Feng J. Li L. Wang R. Cheng H. (2018). Kaempferol protects against gamma radiation-induced mortality and damage via inhibiting oxidative stress and modulating apoptotic molecules in vivo and vitro. *Environ. Toxicol. Pharmacol.* 60 128–137. 10.1016/j.etap.2018.04.014 29705372

[B95] Ward J. H. Jr. (1963). Hierarchical grouping to optimize an objective function. *J. Am. Stat. Assoc.* 58 236–244. 10.1080/01621459.1963.10500845

[B96] Xiao R.-Y. Wu L.-J. Hong X.-X. Tao L. Luo P. Shen X.-C. (2018). Screening of analgesic and anti-inflammatory active component in Fructus Alpiniae zerumbet based on spectrum–effect relationship and GC–MS. *Biomed. Chromatogr.* 32:e4112. 10.1002/bmc.4112 28991393

[B97] Yang Y. Zhang X. Xu M. Wu X. Zhao F. Zhao C. (2018). Quercetin attenuates collagen-induced arthritis by restoration of Th17/Treg balance and activation of Heme Oxygenase 1-mediated anti-inflammatory effect. *Int. Immunopharmacol.* 54 153–162. 10.1016/j.intimp.2017.11.013 29149703

[B98] Yao Z.-H. Yao X. Zhang Y. Zhang S. Hu J. (2018). Luteolin could improve cognitive dysfunction by inhibiting neuroinflammation. *Neurochem. Res.* 43 806–820. 10.1007/s11064-018-2482-2 29392519

[B99] Zager J. J. Lange I. Srividya N. Smith A. Lange B. M. (2019). Gene networks underlying cannabinoid and terpenoid accumulation in *Cannabis*. *Plant Physiol.* 180 1877–1897. 10.1104/pp.18.01506 31138625 PMC6670104

[B100] Zheng Y.-Z. Chen D.-F. Deng G. Guo R. Fu Z.-M. (2018). The surrounding environments on the structure and antioxidative activity of luteolin. *J. Mol. Model.* 24:149. 10.1007/s00894-018-3680-1 29869725

