# Supplementary material for: Identification of Chemotypic Markers in Three Chemotype Categories of Cannabis Using Secondary Metabolites Profiled in Inflorescences, Leaves, Stem Bark, and Roots
Source: Front Plant Sci. 2021 Jul 1;12:699530. doi: 10.3389/fpls.2021.699530 (PMC8283674; doi:10.3389/fpls.2021.699530)
Supplement: Supplementary Figure 1 — Secondary metabolites profiling in cannabis roots, stem bark, leaves, and inflorescences in 82 plants of 21 strains. [file Data_Sheet_1.pdf]

Supplementary Material

Supplementary Table 1. Strain information and assignment of 21 strains into three chemotypes. All strains were provided by licensed cultivator The Emerald Flower Farm Inc. (Kelowna, BC, Canada). Specimens of each variety were stored at the research-licenced Labs-Mart Inc. (Edmonton, AB, Canada).

Variety number	Variety name	Number of plants	Chemotypes	Clusters	"Sativa" or "Indica"	Voucher
**1**	Lemon Garlic OG	4	1-Intermediate	C2	"Indica" dominant	Teff_Lgog
**2**	Royal Medic	3	2-Intermediate	C2	"Sativa" dominant	Teff_Rm
**3**	Blue Hawaiian	4	3-CBD	C1	"Sativa" dominant	Teff_Bh
**4**	Kandy Kush	5	4-CBD	C1	"Sativa" dominant	Teff_KK
**5**	Special	3	5-CBD	C1	Not provided	Teff_Sp
**6**	NN	4	6-CBD	C1	Not provided	Teff_Nn
**7***	Dance World	0	7-Intermediate	N/A	"Sativa" dominant	Teff_Dw
**8**	Treat	4	8-CBD	C1	Not provided	Teff_Tr
**9**	High	5	9-Intermediate	C2	Not provided	Teff_Hi
**10**	CB7	4	10-CBD	C1	Not provided	Teff_Cb
**11**	33°	5	11-THC	C3	Not provided	Teff_33
**12**	Banana Cake	4	12-THC	C3	"Indica" dominant	Teff_BC
**13**	Bananium	5	13-THC	C3	"Indica" dominant	Teff_Bc
**14**	Burmese Blueberry	3	14-THC	C3	"Indica" dominant	Teff_Ba
**15**	Divine Banana	4	15-THC	C3	"Indica" dominant	Teff_Db
**16**	Granddaddy Purple	3	16-THC	C3	"Indica" dominant	Teff_Gp
**17***	Lemon Love	2	17-THC	N/A	"Indica" dominant	Teff_Ll
**18**	Lemon Sorbet	4	18-THC	C3	"Indica" dominant	Teff_Ls
**19**	Meat Head	4	19-THC	C3	"Indica" dominant	Teff_Mh
**20**	Nanitro	4	20-THC	C3	"Indica" dominant	Teff_Na
**21**	Platinum Jelly Punch	4	21-THC	C3	"Indica" dominant	Teff_PJP
**22**	SBSK2 (Lemon Thai)	3	22-THC	C3	50/50 hybrid	Teff_Lt
**23**	Super Sherbet	3	23-THC	C3	"Indica" dominant	Teff_Ss

*Strain 7-intermediate was not included in the final analysis due to unsuccessful rooting. Only two plants were available for strain-17 Lemon Love and they were not included in the analysis.

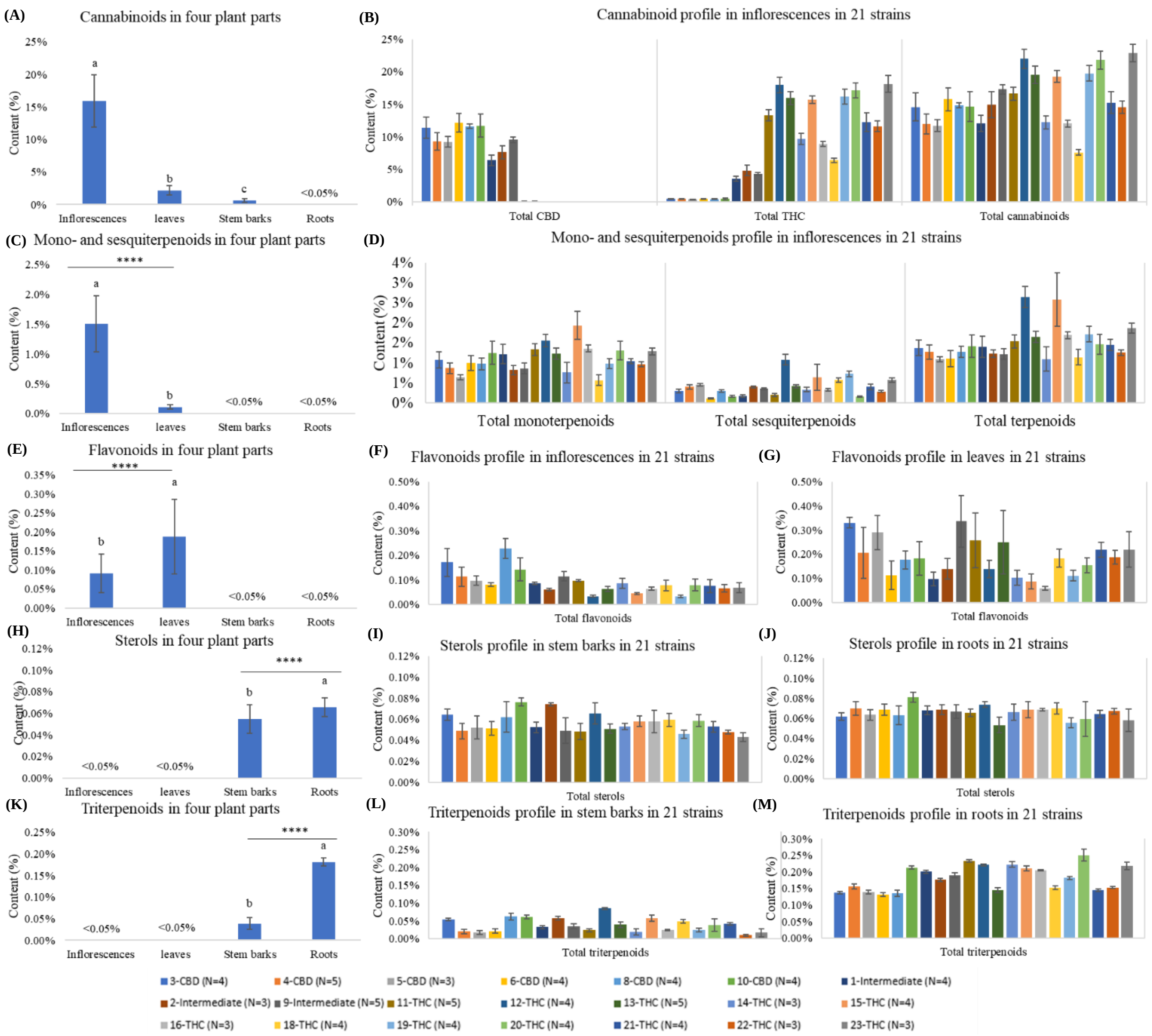

Supplementary Figure 1. Secondary metabolites profiling in cannabis roots, stem barks, leaves, and inflorescences in 82 plants of 21 strains. (A) Total cannabinoid content (mg/mg%) in each plant part averaged from 82 plants (N = 82, mean ± standard deviation (SD)%). **(B)** Total CBD, total THC, and total cannabinoid content (mg/mg%) in inflorescences of 21 strains. **(C)** Total mono- and sesquiterpenoid content (mg/mg%) in each plant part averaged from 82 plants (N = 82, mean ± SD%). **(D)** Total mono- and sesquiterpenoids content (mg/mg%) in inflorescences of 21 strains. **(E)** Total flavonoid content (mg/mg%) in each plant part averaged from 82 plants (N = 82, mean ± SD%). **(F)** Total flavonoid content (mg/mg%) in inflorescences of 21 strains. **(G)** Total flavonoid content (mg/mg%) in leaves of 21 strains. **(H)** Total sterol content (mg/mg%) in each plant part averaged from 82 plants (N = 82, mean ± SD%). **(I)** Total sterol content (mg/mg%) in stem barks of 21 strains. **(J)** Total sterol content (mg/mg%) in roots of 21 strains. **(K)** Total triterpenoid content (mg/mg%) in each plant part averaged from 82 plants (N = 82, mean ± SD%). **(L)** Total triterpenoid content (mg/mg%) in stem barks of 21 strains. **(M)** Total triterpenoid content (mg/mg%) in roots of 21 strains. One-way ANOVA followed by correction for multiple comparisons (Tukey honestly significant difference (HSD) post hoc test) at the 0.05 significance level was used (p values

indicated above each bar). Asterisks indicate statistically significant differences (one-way ANOVA, *p < 0.05, **p < 0.01, ***p < 0.001, ****p < 0.0001).

Supplementary Table 2.1 Secondary metabolites profiled in inflorescences of 82 plants of 21 strains

	Inflorescences (mean ± SD)	Leaves (mean ± SD)	Stem barks (mean ± SD)	Roots (mean ± SD)
**Total cannabinoids**	15.904% ± 4.017%	2.166% ± 0.706%	0.581% ± 0.284%	<0.03%
**Total terpenoids**	1.509% ± 0.467%	0.110% ± 0.037%	<0.03%	<0.03%
**Total flavonoids**	0.091% ± 0.050%	0.188% ± 0.098%	<0.03%	<0.03%
**Total sterols**	<0.03%	<0.03%	0.055% ± 0.013%	0.066% ± 0.009%
**Total triterpenoids**	<0.03%	<0.03%	0.039% ± 0.023%	0.182% ± 0.043%

Supplementary Table 2.2 Cannabinoids profiled in inflorescences of 82 plants for three chemotypes

**Inflorescences**	**C1 - CBD**	**C2 - Intermediate**	**C3 - THC**
	(N=24)	(N=12)	(N=46)
**1. CBDV**	0.0078% ± 0.0004%	0.007% ± 0.003%	0.0002% ± 0.0012%
**2. CBDVA**	0.039% ± 0.010%	0.035% ± 0.013%	0.006% ± 0.006%
**3. CBG**	0.057% ± 0.040%	0.069% ± 0.031%	0.078% ± 0.042%
**4. CBD**	0.374% ± 0.097%	0.216% ± 0.051%	0.005% ± 0.004%
**5. CBDA**	12.020% ± 1.863%	8.931% ± 1.829%	0.061% ± 0.021%
**6. THCV**	0.007% ± 0.002%	0.008% ± 0.000%	0.010% ± 0.003%
**7. CBGA**	0.280% ± 0.102%	0.355% ± 0.219%	0.689% ± 0.402%
**8. CBN**	ND*	ND	ND
**9. Δ^9-THC**	0.042% ± 0.009%	0.294% ± 0.114%	0.350% ± 0.140%
**10. Δ^8-THC**	ND	ND	ND
**11. THCVA**	ND	0.016% ± 0.010%	0.186% ± 0.231%
**12. CBC**	0.040% ± 0.010%	0.027% ± 0.009%	0.017% ± 0.007%
**13. THCA**	0.488% ± 0.088%	4.463% ± 0.808%	15.333% ± 4.221%
**14. CBCA**	0.600% ± 0.140%	0.558% ± 0.143%	0.427% ± 0.224%
**Total CBDV***	0.042% ± 0.009%	0.037% ± 0.010%	0.005% ± 0.006%
**Total CBG***	0.303% ± 0.100%	0.380% ± 0.212%	0.682% ± 0.374%
**Total CBD***	10.915% ± 1.686%	8.049% ± 1.575%	0.059% ± 0.019%
**Total THCV***	0.007% ± 0.002%	0.022% ± 0.009%	0.171% ± 0.203%
**Total THC***	0.471% ± 0.080%	4.208% ± 0.665%	13.797% ± 3.750%
**Total CBC***	0.566% ± 0.127%	0.516% ± 0.120%	0.392% ± 0.200%
**Total cannabinoids***	13.956% ± 2.147%	14.979% ± 2.626%	17.162% ± 4.597%

*Total CBDV = CBDV + 0.867 × CBDVA
*Total CBG = CBG + 0.878 × CBGA.
*Total CBD = CBD + 0.877 × CBDA.
*Total THCV = THCV + 0.867 × THCVA.
*Total THC = Δ^9-THC + Δ^8-THC + CBN + 0.877 × THCA.
*Total CBC = CBC + 0.877× CBCA.
*Total cannabinoids = sum of 14 cannabinoids.
*ND = Not detected or below quantification limit (trace amount).

Supplementary Table 2.3 Cannabinoid profile in leaves of 82 plants for three chemotypes

Leaves	C1 - CBD	C2 - Intermediate	C3 - THC
	(N=18)	(N=9)	(N=43)
**1. CBDV**	0.002% ± 0.004%	0.002% ± 0.004%	ND
**2. CBDVA**	0.021% ± 0.004%	0.0192% ± 0.0004%	0.007% ± 0.009%
**3. CBG**	0.016% ± 0.005%	0.014% ± 0.005%	0.016% ± 0.006%
**4. CBD**	0.070% ± 0.048%	0.043% ± 0.020%	0.008% ± 0.004%
**5. CBDA**	1.438% ± 0.357%	1.012% ± 0.269%	0.056% ± 0.054%
**6. THCV**	0.006% ± 0.018%	0.003% ± 0.005%	0.004% ± 0.005%
**7. CBGA**	0.055% ± 0.021%	0.057% ± 0.025%	0.098% ± 0.068%
**8. CBN**	ND*	ND	ND
**9. Δ^9-THC**	0.019% ± 0.006%	0.063% ± 0.024%	0.141% ± 0.100%
**10. Δ^8-THC**	ND	ND	ND
**11. THCVA**	ND	0.002% ± 0.004%	0.024% ± 0.021%
**12. CBC**	0.020% ± 0.002%	0.022% ± 0.005%	0.032% ± 0.017%
**13. THCA**	0.213% ± 0.114%	0.702% ± 0.165%	1.622% ± 0.621%
**14. CBCA**	0.095% ± 0.019%	0.133% ± 0.037%	0.263% ± 0.180%
**Total CBDV***	0.021% ± 0.005%	0.019% ± 0.005%	0.006% ± 0.008%
**Total CBG***	0.064% ± 0.021%	0.064% ± 0.021%	0.102% ± 0.062%
**Total CBD***	1.332% ± 0.337%	0.931% ± 0.243%	0.057% ± 0.049%
**Total THCV***	0.006% ± 0.018%	0.005% ± 0.005%	0.026% ± 0.021%
**Total THC***	0.206% ± 0.104%	0.680% ± 0.159%	1.564% ± 0.580%
**Total CBC***	0.103% ± 0.016%	0.139% ± 0.032%	0.263% ± 0.166%
**Total cannabinoids**	1.956% ± 0.451%	2.075% ± 0.481%	2.273% ± 0.812%

*Total CBDV = CBDV + 0.867 × CBDVA
*Total CBG = CBG + 0.878 × CBGA.
*Total CBD = CBD + 0.877 × CBDA.
*Total THCV = THCV + 0.867 × THCVA.
*Total THC = Δ^9-THC + Δ^8-THC + CBN + 0.877 × THCA.
*Total CBC = CBC + 0.877× CBCA.
*Total cannabinoids = sum of 14 cannabinoids.
*ND = Not detected or below quantification limit (trace amount).

Supplementary Table 2.4 Cannabinoid profile in stem bark of 82 plants for three chemotypes

Stem barks	C1 - CBD	C2 - Intermediate	C3 - THC
	(N=23)	(N=12)	(N=46)
**1. CBDV**	0.0004% ± 0.0021%	0.001% ± 0.003%	ND
**2. CBDVA**	0.007% ± 0.004%	0.007% ± 0.005%	0.002% ± 0.004%
**3. CBG**	0.009% ± 0.003%	0.0099% ± 0.0001%	0.010% ± 0.001%
**4. CBD**	0.009% ± 0.006%	0.005% ± 0.005%	0.004% ± 0.005%
**5. CBDA**	0.286% ± 0.156%	0.209% ± 0.117%	0.055% ± 0.046%
**6. THCV**	ND*	ND	ND
**7. CBGA**	0.022% ± 0.008%	0.025% ± 0.012%	0.025% ± 0.011%
**8. CBN**	ND	ND	ND
**9. Δ^9-THC**	0.015% ± 0.005%	0.021% ± 0.003%	0.024% ± 0.007%
**10. Δ^8-THC**	ND	ND	ND
**11. THCVA**	0.004% ± 0.005%	0.004% ± 0.005%	0.004% ± 0.007%

**12. CBC**	0.010% ± 0.008%	0.012% ± 0.008%	0.007% ± 0.007%
**13. THCA**	0.150% ± 0.104%	0.219% ± 0.094%	0.446% ± 0.295%
**14. CBCA**	0.032% ± 0.011%	0.030% ± 0.014%	0.030% ± 0.019%
**Total CBDV***	0.007% ± 0.005%	0.007% ± 0.006%	0.001% ± 0.003%
**Total CBG***	0.028% ± 0.008%	0.032% ± 0.011%	0.032% ± 0.009%
**Total CBD***	0.260% ± 0.140%	0.189% ± 0.105%	0.052% ± 0.042%
**Total THCV***	0.004% ± 0.004%	0.004% ± 0.005%	0.004% ± 0.006%
**Total THC***	0.146% ± 0.094%	0.212% ± 0.084%	0.416% ± 0.260%
**Total CBC***	0.038% ± 0.013%	0.037% ± 0.014%	0.034% ± 0.019%
**Total cannabinoids***	0.545% ± 0.252%	0.542% ± 0.232%	0.609% ± 0.312%

*Total CBDV = CBDV + 0.867 × CBDVA
*Total CBG = CBG + 0.878 × CBGA.
*Total CBD = CBD + 0.877 × CBDA.
*Total THCV = THCV + 0.867 × THCVA.
*Total THC = Δ^9-THC + Δ^8-THC + CBN + 0.877 × THCA.
*Total CBC = CBC + 0.877× CBCA.
*Total cannabinoids = sum of 14 cannabinoids.
*ND = Not detected or below quantification limit (trace amount).

Supplementary Table 2.5 Mono- and sesquiterpenoids profile in inflorescences of 82 plants for three chemotypes

**Inflorescences**	**C1 - CBD**	**C2 - Intermediate**	**C3 - THC**
	(N=24)	(N=12)	(N=46)
**1. α-Pinene**	0.187% ± 0.067%	0.083% ± 0.085%	0.130% ± 0.085%
**2. Camphene**	0.006% ± 0.002%	0.007% ± 0.001%	0.015% ± 0.009%
**3. Sabinene**	ND*	ND	ND
**4. β-Pinene**	0.077% ± 0.026%	0.056% ± 0.025%	0.104% ± 0.039%
**5. β-Myrcene**	0.516% ± 0.143%	0.548% ± 0.160%	0.297% ± 0.228%
**6. α-Phellandrene**	ND	ND	ND
**7. Δ^3-Carene**	ND	ND	ND
**8. α-Terpinene**	ND	ND	ND
**9. p-Cymene**	ND	ND	ND
**10. Limonene**	0.092% ± 0.019%	0.134% ± 0.023%	0.326% ± 0.223%
**11. 1,8-Cineole (Eucalyptol)**	0.007% ± 0.002%	0.012% ± 0.007%	0.005% ± 0.005%
**12. Ocimene**	0.015% ± 0.018%	0.009% ± 0.005%	0.077% ± 0.061%
**13. γ-Terpinene**	ND	ND	ND
**14. Sabinene Hydrate**	0.006% ± 0.002%	0.007% ± 0.002%	0.010% ± 0.004%
**15. Terpinolene**	0.008% ± 0.009%	0.012% ± 0.012%	0.063% ± 0.123%
**16. Fenchone**	ND	ND	ND
**17. Linalool**	0.028% ± 0.008%	0.052% ± 0.019%	0.078% ± 0.063%
**18. Fenchol**	0.015% ± 0.003%	0.021% ± 0.004%	0.041% ± 0.028%
**19. (-)-Isopulegol**	ND	ND	ND
**20. Camphor**	ND	ND	ND
**21. Borneol**	0.006% ± 0.001%	0.007% ± 0.001%	0.010% ± 0.005%
**22. Terpinen-4-ol**	ND	ND	ND
**23. α-Terpineol**	0.019% ± 0.004%	0.027% ± 0.004%	0.054% ± 0.027%

**24. (+)-Dihydrocarvone**	ND	ND	ND
**25. Nerol**	ND	ND	ND
**26. Pulegone**	ND	ND	ND
**27. Carvone (isomers)**	ND	ND	ND
**28. Geraniol**	ND	ND	ND
**29. Geranyl Acetate**	ND	ND	ND
**30. (-)-β-Elemene**	ND	ND	ND
**31. β-Caryophyllene**	0.045% ± 0.022%	0.081% ± 0.039%	0.223% ± 0.163%
**32. Aromadendrene**	ND	ND	ND
**33. trans-β-Farnesene**	0.006% ± 0.003%	0.006% ± 0.001%	0.025% ± 0.028%
**34. α-Humulene**	0.014% ± 0.007%	0.024% ± 0.012%	0.075% ± 0.048%
**35. Valencene**	ND	ND	ND
**36. Ledene**	ND	ND	ND
**37. trans-Nerolidol**	0.006% ± 0.002%	0.006% ± 0.002%	0.052% ± 0.048%
**38. Caryophyllene Oxide**	ND	ND	ND
**39. Globulol**	ND	ND	ND
**40. Viridiflorol**	ND	ND	ND
**41. (-)-Guaiol**	0.062% ± 0.025%	0.063% ± 0.023%	0.028% ± 0.026%
**42.(+)-Cedrol**	ND	ND	ND
**43. β-Eudesmol**	0.036% ± 0.015%	0.034% ± 0.011%	0.015% ± 0.015%
**44. α-Eudesmol**	0.021% ± 0.009%	0.018% ± 0.005%	0.010% ± 0.009%
**45. α-Bisabolol**	0.100% ± 0.061%	0.080% ± 0.028%	0.053% ± 0.030%
**Total monoterpenoids***	0.980% ± 0.243%	0.974% ± 0.241%	1.211% ± 0.383%
**Total sesquiterpenoids***	0.289% ± 0.124%	0.311% ± 0.099%	0.482% ± 0.275%
**Total terpenoids**	1.269% ± 0.209%	1.285% ± 0.187%	1.693% ± 0.532%

*Total monoterpenoids = sum of terpenes 1 – 29.
*Total sesquiterpenoids = sum of terpenes 30 – 45.
*ND = Not detected or below quantification limit (trace amount).

Supplementary Table 2.6 Mono- and sesquiterpenoids profile in leaves of 82 plants for three chemotypes

**Leaves**	**C1 - CBD**	**C2 - Intermediate**	**C3 - THC**
	(N=18)	(N=9)	(N=43)
**1. α-Pinene**	0.010% ± 0.006%	0.007% ± 0.006%	0.004% ± 0.004%
**2. Camphene**	ND	ND	ND
**3. Sabinene**	ND	ND	ND
**4. β-Pinene**	0.003% ± 0.002%	0.003% ± 0.001%	0.003% ± 0.002%
**5. β-Myrcene**	0.008% ± 0.007%	0.003% ± 0.001%	0.004% ± 0.004%
**6. α-Phellandrene**	ND	ND	ND
**7. Δ^3-Carene**	ND	ND	ND
**8. α-Terpinene**	ND	ND	ND
**9. p-Cymene**	ND	ND	ND
**10. Limonene**	0.002% ± 0.001%	0.002% ± 0.001%	0.004% ± 0.003%
**11. 1,8-Cineole (Eucalyptol)**	0.002% ± 0.001%	0.003% ± 0.002%	0.003% ± 0.005%

**12. Ocimene**	ND	ND	ND
**13. γ-Terpinene**	ND	ND	ND
**14. Sabinene Hydrate**	ND	ND	ND
**15. Terpinolene**	ND	ND	ND
**16. Fenchone**	ND	ND	ND
**17. Linalool**	0.001% ± 0.0004%	0.002% ± 0.0005%	0.002% ± 0.002%
**18. Fenchol**	0.001% ± 0.001%	0.001% ± 0.000%	0.002% ± 0.001%
**19. (-)-Isopulegol**	ND	ND	ND
**20. Camphor**	ND	ND	ND
**21. Borneol**	ND	ND	ND
**22. Terpinen-4-ol**	ND	ND	ND
**23. α-Terpineol**	ND	ND	ND
**24. (+)-Dihydrocarvone**	ND	ND	ND
**25. Nerol**	ND	ND	ND
**26. Pulegone**	ND	ND	ND
**27. Carvone (isomers)**	ND	ND	ND
**28. Geraniol**	ND	ND	ND
**29. Geranyl Acetate**	ND	ND	ND
**30. (-)-β-Elemene**	ND	ND	ND
**31. β-Caryophyllene**	0.012% ± 0.005%	0.015% ± 0.007%	0.027% ± 0.012%
**32. Aromadendrene**	ND	ND	ND
**33. trans-β-Farnesene**	0.003% ± 0.001%	0.003% ± 0.0004%	0.005% ± 0.003%
**34. α-Humulene**	0.004% ± 0.001%	0.005% ± 0.002%	0.009% ± 0.005%
**35. Valencene**	ND	ND	ND
**36. Ledene**	ND	ND	ND
**37. trans-Nerolidol**	0.001% ± 0.0004%	0.001% ± 0.0004%	0.003% ± 0.002%
**38. Caryophyllene Oxide**	ND	ND	ND
**39. Globulol**	ND	ND	ND
**40. Viridiflorol**	ND	ND	ND
**41. (-)-Guaiol**	0.010% ± 0.005%	0.012% ± 0.002%	0.004% ± 0.004%
**42.(+)-Cedrol**	ND	ND	ND
**43. β-Eudesmol**	0.007% ± 0.004%	0.008% ± 0.002%	0.003% ± 0.002%
**44. α-Eudesmol**	0.005% ± 0.002%	0.006% ± 0.001%	0.002% ± 0.002%
**45. α-Bisabolol**	0.036% ± 0.020%	0.045% ± 0.013%	0.023% ± 0.013%
**Total monoterpenoids**	0.027% ± 0.016%	0.021% ± 0.008%	0.021% ± 0.011%
**Total sesquiterpenoids**	0.077% ± 0.035%	0.094% ± 0.020%	0.077% ± 0.026%
**Total terpenoids**	0.104% ± 0.048%	0.115% ± 0.019%	0.099% ± 0.029%

*Total monoterpenoids = sum of terpenoids 1 – 29.
*Total sesquiterpenoids = sum of terpenoids 30 – 45.
*ND = Not detected or below quantification limit (trace amount).

Supplementary Table 2.7 Flavonoids profile in inflorescences for three chemotypes

**Inflorescences**	**C1 - CBD**	**C2 - Intermediate**	**C3 - THC**
	(N=24)	(N=12)	(N=46)

**1. Orientin (F)**	0.051% ± 0.022%	0.021% ± 0.008%	0.014% ± 0.012%
**2. Vitexin (F)**	0.042% ± 0.018%	0.024% ± 0.006%	0.013% ± 0.011%
**3. Isovitexin (F)**	0.003% ± 0.001%	0.002% ± 0.001%	0.001% ± 0.001%
**4. Quercetin (F)**	0.008% ± 0.004%	0.014% ± 0.008%	0.012% ± 0.006%
**5. Luteolin (F)**	0.027% ± 0.023%	0.021% ± 0.006%	0.018% ± 0.021%
**6. Kaempferol (F)**	0.0030% ± 0.0004%	0.003% ± 0.001%	0.004% ± 0.001%
**7. Apigenin (F)**	0.006% ± 0.004%	0.007% ± 0.001%	0.003% ± 0.002%
**Total flavonoids**	0.140% ± 0.061%	0.092% ± 0.026%	0.065% ± 0.025%

Note: Flavonoids in inflorescences is labelled (F).

Supplementary Table 2.8 Flavonoids profile in leaves for three chemotypes

**Leaves**	**C1 - CBD**	**C2 - Intermediate**	**C3 - THC**
	(N=24)	(N=12)	(N=46)
**1. Orientin (L)**	0.077% ± 0.064%	0.044% ± 0.032%	0.038% ± 0.039%
**2. Vitexin (L)**	0.061% ± 0.036%	0.053% ± 0.036%	0.032% ± 0.026%
**3. Isovitexin (L)**	0.004% ± 0.003%	0.004% ± 0.003%	0.002% ± 0.002%
**4. Quercetin (L)**	ND	ND	ND
**5. Luteolin (L)**	0.050% ± 0.040%	0.074% ± 0.046%	0.074% ± 0.068%
**6. Kaempferol (L)**	ND	ND	ND
**7. Apigenin (L)**	0.017% ± 0.012%	0.021% ± 0.008%	0.016% ± 0.011%
**Total flavonoids**	0.213% ± 0.095%	0.208% ± 0.134%	0.170% ± 0.087%

Note: Flavonoids in leaves is labelled (L).

Supplementary Table 2.9 Sterols profile in stem bark for three chemotypes

**Stem barks**	**C1 - CBD**	**C2 - Intermediate**	**C3 - THC**
	(N=24)	(N=12)	(N=46)
**1. Campesterol**	0.012% ± 0.002%	0.012% ± 0.004%	0.011% ± 0.003%
**2. Stigmasterol**	0.010% ± 0.003%	0.010% ± 0.003%	0.011% ± 0.004%
**3. β-sitosterol**	0.037% ± 0.008%	0.034% ± 0.008%	0.030% ± 0.008%
**Total sterols**	0.059% ± 0.012%	0.056% ± 0.013%	0.052% ± 0.014%

Supplementary Table 2.10 Sterols profile in roots for three chemotypes

**Roots**	**C1 - CBD**	**C2 - Intermediate**	**C3 - THC**
	(N=24)	(N=12)	(N=46)
**1. Campesterol**	0.013% ± 0.001%	0.013% ± 0.001%	0.012% ± 0.002%
**2. Stigmasterol**	0.012% ± 0.002%	0.013% ± 0.001%	0.013% ± 0.002%
**3. β-sitosterol**	0.043% ± 0.006%	0.042% ± 0.004%	0.039% ± 0.007%
**Total sterols**	0.068% ± 0.009%	0.068% ± 0.005%	0.064% ± 0.009%

Supplementary Table 2.11 Triterpenoids profile in stem bark for three chemotypes

**Stem barks**	**C1 - CBD**	**C2 - Intermediate**	**C3 - THC**
	(N=24)	(N=12)	(N=46)
**1. β-Amyrin**	0.015% ± 0.007%	0.021% ± 0.005%	0.015% ± 0.008%

**2. Epifriedanol**	0.011% ± 0.008%	0.007% ± 0.004%	0.008% ± 0.008%
**3. Friedelin**	0.014% ± 0.009%	0.012% ± 0.009%	0.015% ± 0.015%
**Total triterpenoids**	0.040% ± 0.022%	0.040% ± 0.013%	0.038% ± 0.025%

Supplementary Table 2.12 Triterpenoids profile in roots for three chemotypes

**Roots**	**C1 - CBD**	**C2 - Intermediate**	**C3 - THC**
	(N=24)	(N=12)	(N=46)
**1. β-Amyrin**	0.004% ± 0.001%	0.006% ± 0.001%	0.006% ± 0.001%
**2. Epifriedanol**	0.055% ± 0.010%	0.064% ± 0.005%	0.062% ± 0.014%
**3. Friedelin**	0.094% ± 0.024%	0.120% ± 0.011%	0.127% ± 0.034%
**Total triterpenoids**	0.153% ± 0.032%	0.190% ± 0.016%	0.194% ± 0.046%

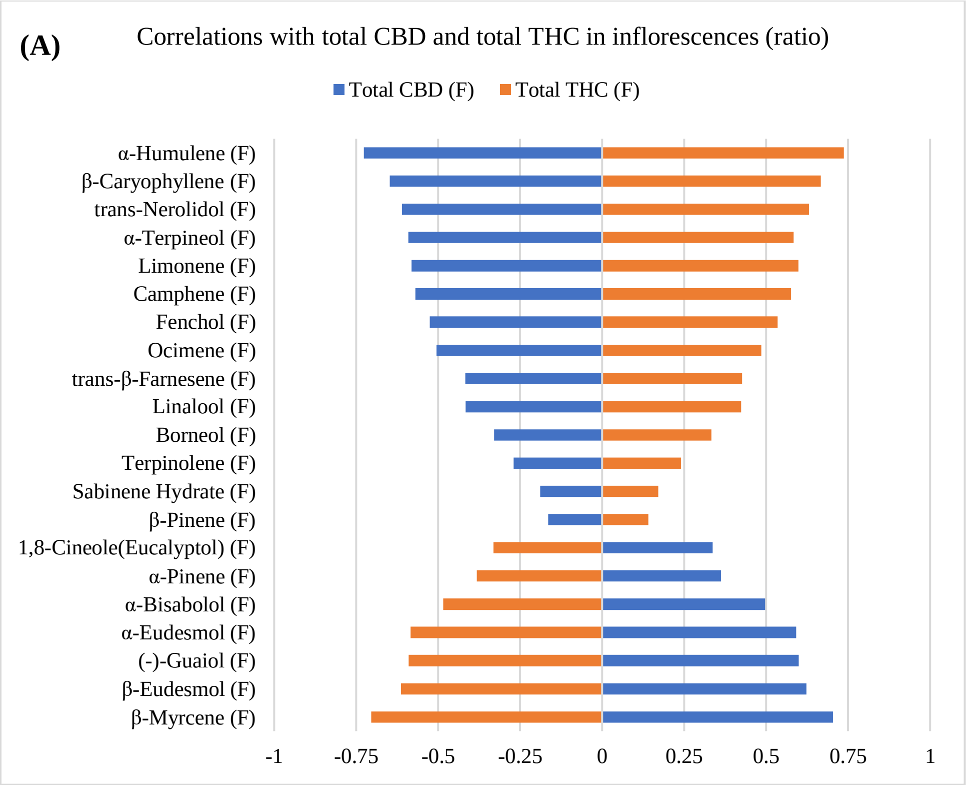

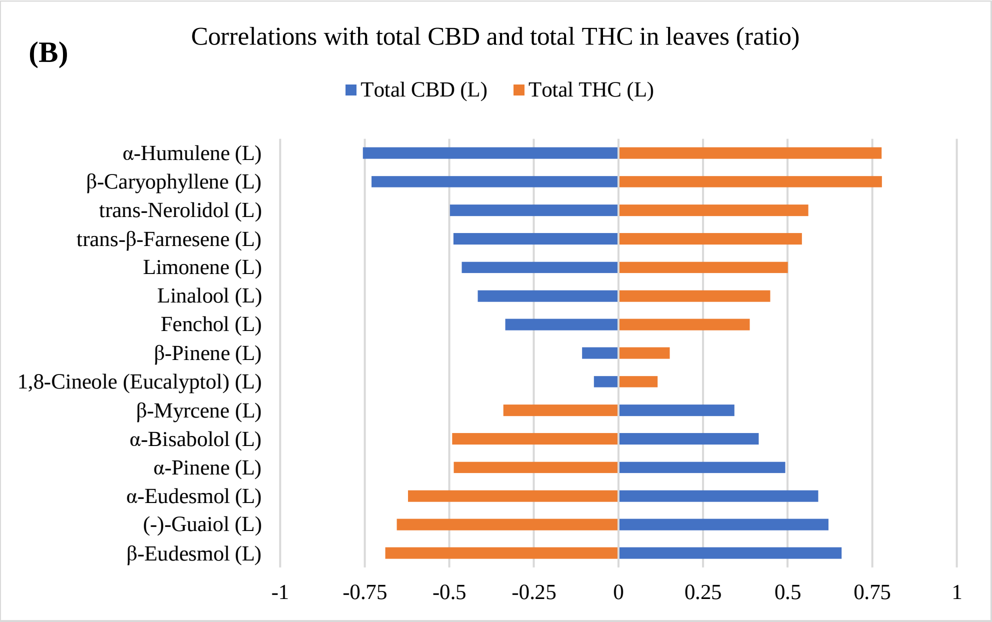

Supplementary Figure 2. Correlations of total THC and total CBD with terpenoids using content ratios (%/%) in (A) inflorescences and in (B) leaves. Compound quantified in inflorescences are labelled as (F). Compound quantified in leaves are labelled as (L).

Supplementary Table 3. Correlations of total THC and total CBD with minor cannabinoids (in inflorescences), mono- and sesquiterpenoids (in inflorescences), flavonoids (in inflorescences and leaves), sterols and triterpenoids (in roots) (only positive correlations are shown)

		**Correlations with total THC**		**Correlations with total CBD**
**Cannabinoids**	Total THCV	0.48	Total CBDV	0.91
	Total CBG	0.38	Total CBC	0.70
**Monoterpenoids**	Limonene	0.59	β-Myrcene	0.71
	α-Terpineol	0.58	α-Pinene	0.38
	Camphene	0.57	1,8-Cineole (eucalyptol)	0.36
	Fenchol	0.52		
	Ocimene	0.49		
	Linalool	0.41		
	Borneol	0.31		
	Terpinolene	0.24		
	Sabinene hydrate	0.15		
	β-Pinene	0.11		
**Sesquiterpenoids**	α-Humulene	0.73	β-Eudesmol	0.63
	β-Caryophyllene	0.66	(-)-Guaiol	0.61
	trans-Nerolidol	0.63	α-Eudesmol	0.60
	trans-β-Farnesene	0.42	α-Bisabolol	0.51
**Flavonoids***	Quercetin (F)	0.70	Orientin (F)	0.48

	Kaempferol (F)	0.59	Vitexin (F)	0.42
	Luteolin (L)	0.31	Isovitexin (F)	0.36
	Luteolin (F)	0.26	Vitexin (L)	0.25
	Apigenin (F)	0.14	Orientin (L)	0.23
	Apigenin (L)	0.07	Isovitexin (L)	0.06
**Sterols**			β-sitosterol	0.50
			Campesterol	0.44
			Stigmasterol	0.23
**Triterpenoids**	Friedelin	0.38	Epifriedanol	0.40
	β-Amyrin	0.28		

Note: Flavonoids in inflorescences is labelled (F), and flavonoids in leaves is labelled (L).

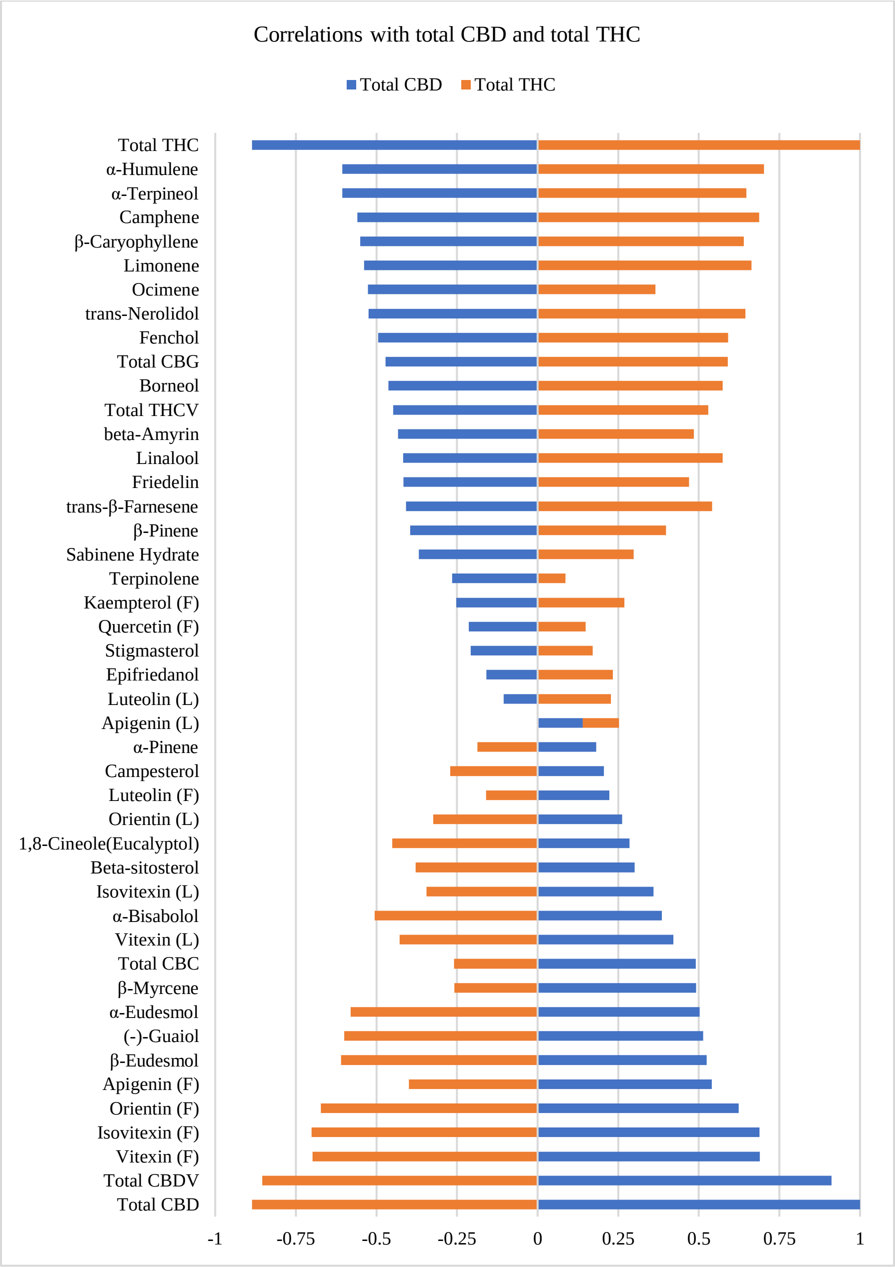

Supplementary Figure 3. Correlations of total THC and total CBD with cannabinoids (in inflorescences), mono- and sesquiterpenoids (in inflorescences), flavonoids (in inflorescences and leaves), sterols and triterpenoids (in roots) on quantifiable compounds using absolute values. Flavonoids quantified in inflorescences is labelled (F), and flavonoids in leaf is labelled (L).

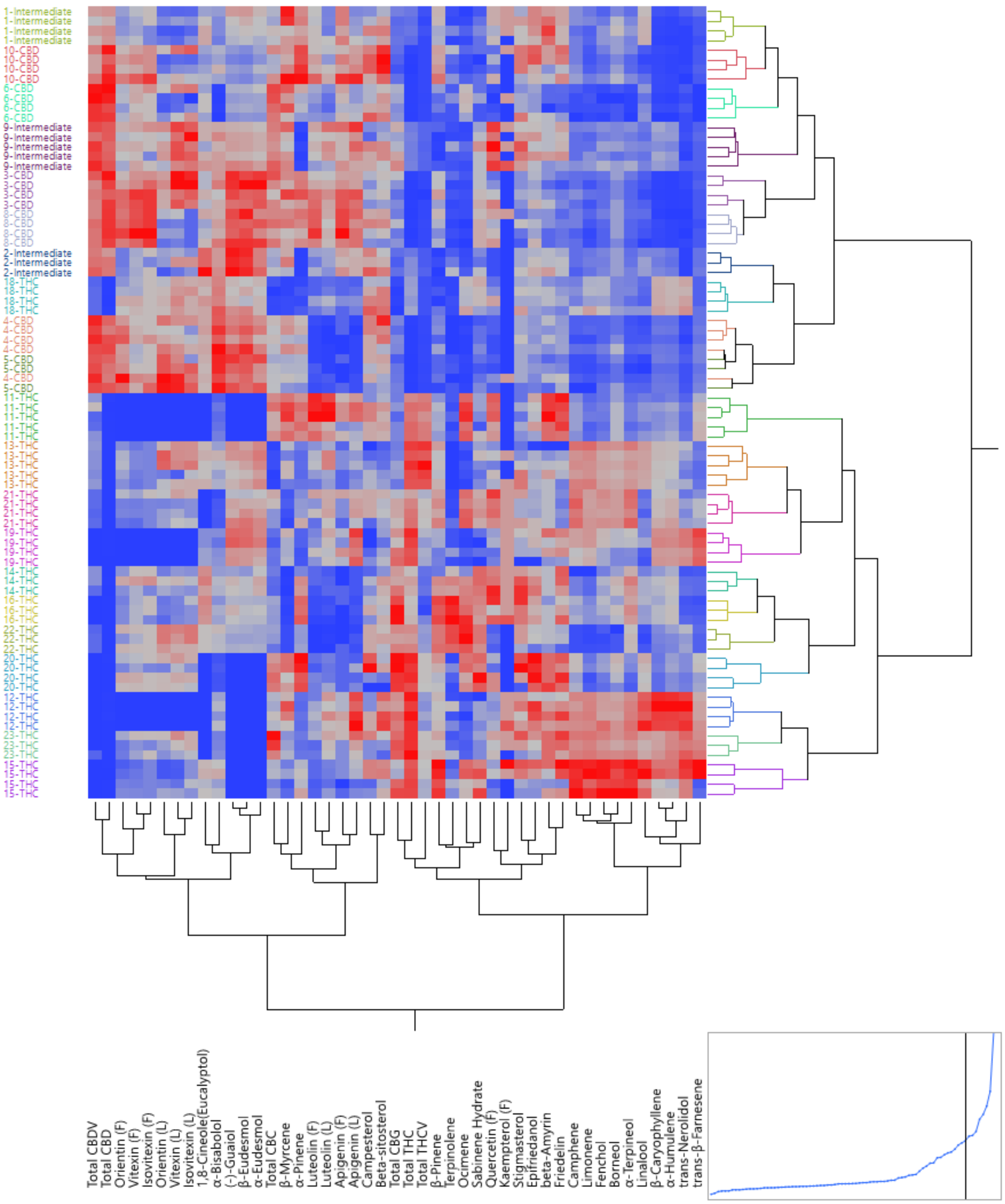

Supplementary Figure 4. Dendrogram by hierarchical clustering analysis using the full spectrum of secondary metabolites (absolute values) of 82 plants representing 21 strains.

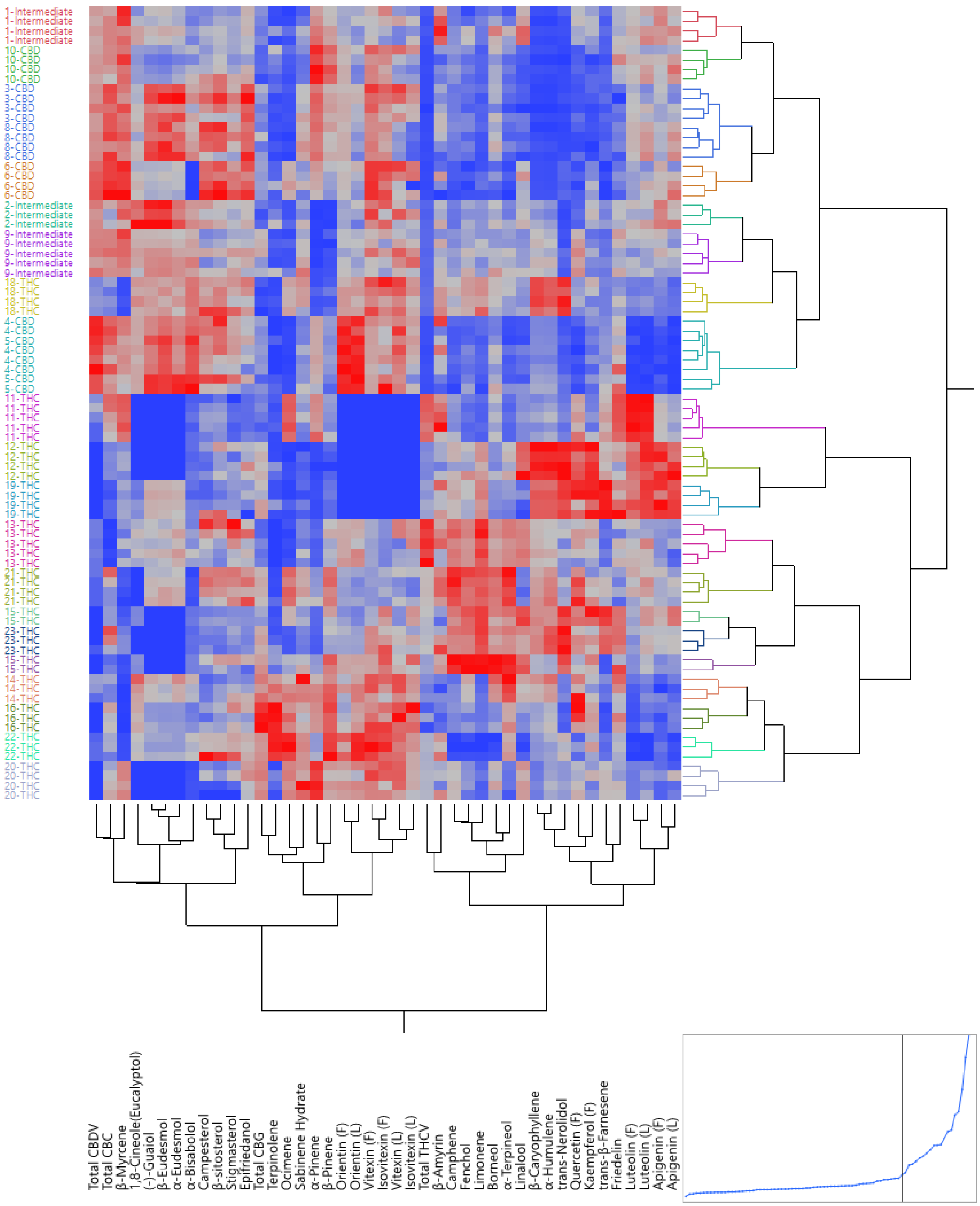

Supplementary Figure 5. Dendrogram by hierarchical clustering analysis using the full spectrum of secondary metabolites (using ratios) without total THC and total CBD.

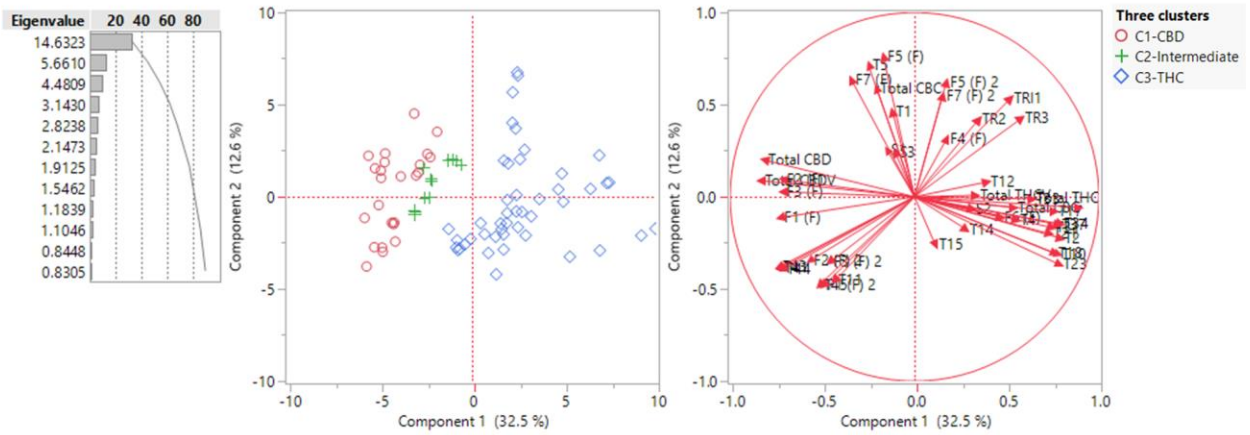

Supplementary Figure 6. PCA scatter plot (left) and loading plot (right) using the full spectrum of secondary metabolites (absolute values) of 82 plants representing 21 strains. Terpenoids are labelled with T and the number assigned in **Supplementary Table 2.5**. Flavonoids are labelled as F and the number assigned in **Supplementary Table 2.7**. Flavonoids quantified in inflorescences are labelled (F) and flavonoids in leaf are labelled (L). Sterols are labelled as S and the number assigned in **Supplementary Table 2.9**. Triterpenoids are labelled as TRI and the number assigned in **Supplementary Table 2.11**.

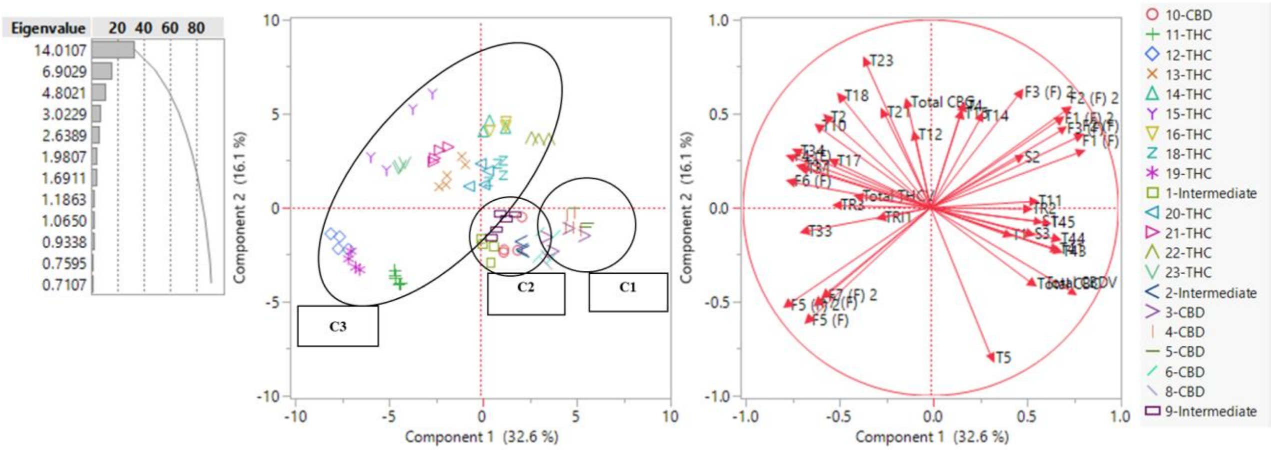

Supplementary Figure 7. PCA scatter plot (left) and loading plot (right) using the full spectrum of secondary metabolites (using ratios) without total THC and total CBD. Terpenoids are labelled with T and the number assigned in **Supplementary Table 2.5**. Flavonoids are labelled as F and the number assigned in **Supplementary Table 2.7**. Flavonoids quantified in inflorescences are labelled (F) and flavonoids in leaf are labelled (L). Sterols are labelled as S and the number assigned in **Supplementary Table 2.9**. Triterpenoids are labelled as TRI and the number assigned in **Supplementary Table 2.11**.

Supplementary Table 4 Summary prediction of 82 plants into preassigned chemotypes using canonical correlation analysis (using ratios)

**Preassigned**	**Predicted**		
	**C1-CBD**	**C2-Intermediate**	**C3-THC**
**C1-CBD**	24	0	0
**C2-Intermediate**	0	12	0
**C3-THC**	0	0	46

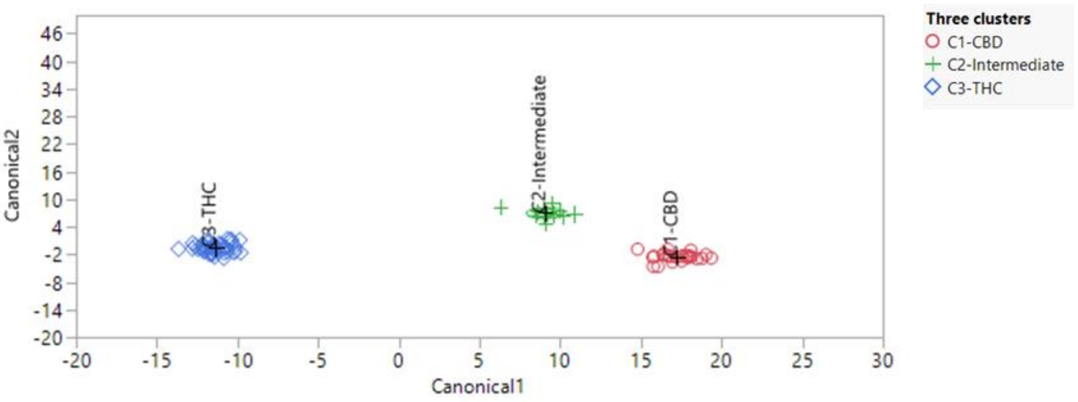

Supplementary Figure 8. Canonical correlation analysis using the full spectrum of secondary metabolites (absolute values) of 82 plants representing 21 strains. The plants were preassigned to three chemotypes in **Table 1**. The observations and the multivariate means of each group (“+”) are represented as points on the biplot. An ellipse denoting a 50% contour is plotted for each group, that contains approximately 50% of the observations.

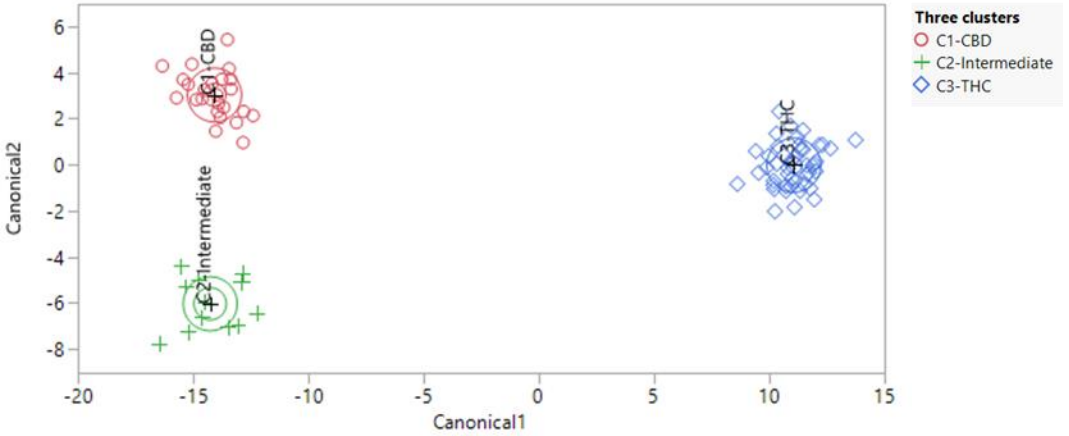

Supplementary Figure 9. Canonical correlation analysis using the full spectrum of secondary metabolites (using ratios) without total THC and total CBD of 82 plants representing 21 strains. The plants were preassigned to three chemotypes in **Table 1**. The observations and the multivariate means of each group (“+”) are represented as points on the biplot. A 95% confidence level ellipse is plotted for each mean. An ellipse denoting a 50% contour is plotted for each group, that contains approximately 50% of the observations.

Supplementary Table 5. Means (±SD) of the ratios of 45 secondary metabolites above quantification limit for 82 plants assigned to C1-CBD dominant, C2-intermediate, and C3-THC dominant.

	**Three chemotypes**	**C1-CBD**	**C2-Intermediate**	**C3-THC**	**ANOVA**
	Plant count	N=24	N=12	N=46	p
**Cannabinoids**	Total CBDV	0.31% ± 0.09% a	0.24% ± 0.04% b	0.04% ± 0.04% c	<0.0001
	Total CBG	2.16% ± 0.55% b	2.45% ± 1.03% b	3.99% ± 2.41% a	0.0004
	Total CBD	78.20% ± 1.27% a	53.58% ± 2.17% b	0.37% ± 0.16% c	<0.0001
	Total THCV	5.42% ± 0.02% b	0.14% ± 0.04% b	0.94% ± 1.05% a	<0.0001
	Total THC	3.40% ± 0.49% c	28.38% ± 3.40% b	80.39% ± 2.41% a	<0.0001
	Total CBC	4.04% ± 0.52% a	3.45% ± 0.46% b	2.31% ± 0.97% c	<0.0001
**Monoterpenoids**	α-Pinene	14.41% ± 3.82% a	6.15% ± 6.01% b	8.10% ± 5.72% b	<0.0001
	Camphene	0.43% ± 0.08% b	0.51% ± 0.05% b	0.86% ± 0.40% a	<0.0001
	β-Pinene	5.94% ± 1.39% a	4.25% ± 1.61% b	6.16% ± 2.03% a	0.0067
	β-Myrcene	40.09% ± 7.23% a	41.53% ± 6.21% a	17.12% ± 13.39% b	<0.0001
	Limonene	7.18% ± 0.77% b	10.43% ± 1.60% b	17.88% ± 9.41% a	<0.0001
	1,8-Cineole (Eucalyptol)	0.58% ± 0.17% b	0.94% ± 0.60% a	0.33% ± 0.38% c	<0.0001
	Ocimene	1.22% ± 1.61% b	0.77% ± 0.44% b	4.89% ± 3.94% a	<0.0001
	Sabinene Hydrate	0.50% ± 0.18% a	0.55% ± 0.24% a	0.62% ± 0.38% a	0.3066
	Terpinolene	0.64% ± 0.70% a	1.03% ± 1.05% a	3.94% ± 7.51% a	0.0482
	Linalool	2.23% ± 0.86% b	4.00% ± 1.42% a	4.11% ± 2.05% a	0.0001
	Fenchol	1.16% ± 0.21% b	1.65% ± 0.41% ab	2.29% ± 1.10% a	<0.0001
	Borneol	0.47% ± 0.09% b	0.53% ± 0.11% ab	0.58% ± 0.20% a	0.0179
	α-Terpineol	1.48% ± 0.26% b	2.09% ± 0.42% b	3.09% ± 1.31% a	<0.0001
**Sesquiterpenoids**	β-Caryophyllene	3.63% ± 1.95% b	6.47% ± 3.28% b	12.27% ± 0.60% a	<0.0001
	trans-β-Farnesene	0.47% ± 0.23% b	0.50% ± 0.12% b	1.34% ± 1.23% a	0.0005
	α-Humulene	1.12% ± 0.65% b	1.91% ± 1.00% b	4.16% ± 1.69% a	<0.0001
	trans-Nerolidol	0.45% ± 0.18% b	0.44% ± 0.14% b	2.75% ± 1.95% a	<0.0001
	(-)-Guaiol	4.90% ± 2.02% a	4.94% ± 2.05% a	1.89% ± 1.76% b	<0.0001
	β-Eudesmol	2.83% ± 1.18% a	2.72% ± 0.99% a	1.04% ± 0.97% b	<0.0001
	α-Eudesmol	1.67% ± 0.71% a	1.40% ± 0.44% a	0.66% ± 0.62% b	<0.0001
	α-Bisabolol	8.01% ± 5.28% a	6.34% ± 2.59% a	3.40% ± 2.62% b	<0.0001
**Flavonoids**	Orientin (F)	38.61% ± 14.12% a	22.48% ± 2.63% b	20.31% ± 14.42% b	<0.0001
	Vitexin (F)	30.41% ± 3.87% a	26.72% ± 4.03% ab	19.50% ± 13.80% b	0.0004
	Isovitexin (F)	2.44% ± 0.44% a	1.97% ± 0.57% ab	1.61% ± 1.76% b	0.0034
	Quercetin (F)	5.42% ± 1.17% c	14.25% ± 5.35% b	19.81% ± 7.54% a	<0.0001
	Luteolin (F)	16.64% ± 9.76% a	22.96% ± 4.07% a	26.69% ± 21.34% a	0.0698
	Kaempferol (F)	2.49% ± 0.97% b	4.01% ± 0.89% b	6.53% ± 3.35% a	<0.0001
	Apigenin (F)	3.99% ± 1.92% b	7.61% ± 2.15% a	5.57% ± 4.25% ab	0.0144
	Orientin (L)	33.16% ± 19.50% a	20.22% ± 4.43% ab	22.45% ± 17.71% b	0.029
	Vitexin (L)	27.64% ± 8.80% a	23.88% ± 8.41% a	20.18% ± 15.02% a	0.0689
	Isovitexin (L)	1.81% ± 0.98% a	1.91% ± 0.83% a	1.68% ± 1.32% a	0.8075
	Luteolin (L)	26.08% ± 18.61% b	37.59% ± 8.40% ab	41.67% ± 24.33% a	0.017

	Apigenin (L)	9.66% ± 7.72% a	12.08% ± 4.49% a	11.04% ± 9.22% a	0.6795
**Sterols**	Campesterol	8.64% ± 1.36% a	6.71% ± 1.08% b	6.56% ± 1.88% b	<0.0001
	Stigmasterol	8.04% ± 1.70% a	7.13% ± 0.80% ab	7.11% ± 1.80% b	0.0494
	β-sitosterol	28.98% ± 4.92% a	22.00% ± 2.52% b	21.09% ± 5.99% b	<0.0001
**Triterpenoids**	β-Amyrin	2.63% ± 0.37% b	3.02% ± 0.38% a	2.92% ± 0.40% a	0.0045
	Epifriedanol	36.60% ± 4.88% a	33.89% ± 1.44% ab	32.12% ± 4.54% b	0.0005
	Friedelin	60.78% ± 4.82% b	63.08% ± 1.55% ab	64.96% ± 4.52% a	0.0011

*Levels not connected by same letter are significantly different.

Supplementary Table 6. Means (±SD) of the absolute values of 45 secondary metabolites (mg/mg%) for 82 plants assigned to C1-CBD dominant, C2-intermediate, and C3-THC dominant.

	**Three chemotypes**	**C1-CBD**	**C2-Intermediate**	**C3-THC**	**ANOVA**
	Plant count	N=24	N=12	N=46	p
**Cannabinoids**	Total CBDV	0.042% ± 0.009% a	0.037% ± 0.010% a	0.005% ± 0.006% b	<0.0001
	Total CBG	0.303% ± 0.100% b	0.380% ± 0.212% b	0.682% ± 0.374% a	<0.0001
	Total CBD	10.915% ± 1.686% a	8.049% ± 1.575% b	0.059% ± 0.019% c	<0.0001
	Total THCV	0.007% ± 0.002% b	0.022% ± 0.009% b	0.171% ± 0.203% a	<0.0001
	Total THC	0.471% ± 0.080% c	4.208% ± 0.665% b	13.797% ± 3.750% a	<0.0001
	Total CBC	0.566% ± 0.127% a	0.516% ± 0.120% ab	0.392% ± 0.200% a	0.0003
**Monoterpenoids**	α-Pinene	0.187% ± 0.067% a	0.083% ± 0.085% b	0.130% ± 0.085% b	0.0010
	Camphene	0.006% ± 0.002% b	0.007% ± 0.001% b	0.015% ± 0.009% a	<0.0001
	β-Pinene	0.077% ± 0.026% b	0.056% ± 0.025% b	0.104% ± 0.039% a	<0.0001
	β-Myrcene	0.516% ± 0.143% a	0.548% ± 0.160% a	0.297% ± 0.228% b	<0.0001
	Limonene	0.092% ± 0.019% b	0.134% ± 0.023% b	0.326% ± 0.223% a	<0.0001
	1,8-Cineole (Eucalyptol)	0.007% ± 0.002% b	0.012% ± 0.007% a	0.005% ± 0.005% b	<0.0001
	Ocimene	0.015% ± 0.018% b	0.009% ± 0.005% b	0.077% ± 0.061% a	<0.0001
	Sabinene Hydrate	0.006% ± 0.002% b	0.007% ± 0.002% b	0.010% ± 0.004% a	0.0007
	Terpinolene	0.008% ± 0.009% a	0.012% ± 0.012% a	0.063% ± 0.123% a	0.0411
	Linalool	0.028% ± 0.008% b	0.052% ± 0.019% ab	0.078% ± 0.063% a	0.0003
	Fenchol	0.015% ± 0.003% b	0.021% ± 0.004% b	0.041% ± 0.028% a	<0.0001
	Borneol	0.006% ± 0.001% b	0.007% ± 0.001% b	0.010% ± 0.005% a	<0.0001
	α-Terpineol	0.019% ± 0.004% b	0.027% ± 0.004% b	0.054% ± 0.027% a	<0.0001
**Sesquiterpenoids**	β-Caryophyllene	0.045% ± 0.022% b	0.081% ± 0.039% b	0.223% ± 0.163% a	<0.0001
	trans-β-Farnesene	0.006% ± 0.003% b	0.006% ± 0.001% b	0.025% ± 0.028% a	0.0006
	α-Humulene	0.014% ± 0.007% b	0.024% ± 0.012% b	0.075% ± 0.048% a	<0.0001
	trans-Nerolidol	0.006% ± 0.002% b	0.006% ± 0.002% b	0.052% ± 0.048% a	<0.0001
	(-)-Guaiol	0.062% ± 0.025% a	0.063% ± 0.023% a	0.028% ± 0.026% b	<0.0001
	β-Eudesmol	0.036% ± 0.015% a	0.034% ± 0.011% a	0.015% ± 0.015% b	<0.0001
	α-Eudesmol	0.021% ± 0.009% a	0.018% ± 0.005% a	0.010% ± 0.009% b	<0.0001
	α-Bisabolol	0.100% ± 0.061% a	0.080% ± 0.028% ab	0.053% ± 0.030% b	<0.0001
**Flavonoids**	Orientin (F)	0.051% ± 0.022% a	0.021% ± 0.008% b	0.014% ± 0.012% b	<0.0001

	Vitexin (F)	0.042% ± 0.018% a	0.024% ± 0.006% b	0.013% ± 0.011% c	<0.0001
	Isovitexin (F)	0.003% ± 0.001% a	0.002% ± 0.001% b	0.001% ± 0.001% b	<0.0001
	Quercetin (F)	0.008% ± 0.004% b	0.014% ± 0.008% a	0.012% ± 0.006% a	0.0012
	Luteolin (F)	0.027% ± 0.023% a	0.021% ± 0.006% a	0.018% ± 0.021% a	0.2290
	Kaempferol (F)	0.0030% ± 0.0004% b	0.003% ± 0.001% ab	0.004% ± 0.001% a	0.0156
	Apigenin (F)	0.006% ± 0.004% a	0.007% ± 0.001% a	0.003% ± 0.002% b	<0.0001
	Orientin (L)	0.077% ± 0.064% a	0.044% ± 0.032% ab	0.038% ± 0.039% b	0.0061
	Vitexin (L)	0.061% ± 0.036% a	0.053% ± 0.036% ab	0.032% ± 0.026% b	0.0010
	Isovitexin (L)	0.004% ± 0.003% a	0.004% ± 0.003% ab	0.002% ± 0.002% b	0.0098
	Luteolin (L)	0.050% ± 0.040% a	0.074% ± 0.046% a	0.074% ± 0.068% a	0.2586
	Apigenin (L)	0.017% ± 0.012% a	0.021% ± 0.008% a	0.016% ± 0.011% a	0.5547
**Sterols**	Campesterol	0.013% ± 0.001% a	0.013% ± 0.001% a	0.012% ± 0.002% a	0.1279
	Stigmasterol	0.012% ± 0.002% b	0.013% ± 0.001% ab	0.013% ± 0.002% a	0.0361
	β-Sitosterol	0.043% ± 0.006% a	0.042% ± 0.004% ab	0.039% ± 0.007% b	0.0169
**Triterpenoids**	β-Amyrin	0.004% ± 0.001% b	0.006% ± 0.001% a	0.006% ± 0.001% a	<0.0001
	Epifriedanol	0.055% ± 0.010% a	0.064% ± 0.005% a	0.062% ± 0.014% a	0.0477
	Friedelin	0.094% ± 0.024% b	0.120% ± 0.011% a	0.127% ± 0.034% a	0.0001

*Levels not connected by same letter are significantly different.
